# ﻿An RNA aptamer that shifts the reduction potential of metabolic cofactors

**DOI:** 10.1038/s41589-022-01121-4

**Published:** 2022-09-12

**Authors:** John S. Samuelian, Thomas J. Gremminger, Zhenwei Song, Raghav R. Poudyal, Jun Li, Yuanzhe Zhou, Seth A. Staller, Johan A. Carballo, Manami Roychowdhury-Saha, Shi-Jie Chen, Donald H. Burke, Xiao Heng, Dana A. Baum

**Affiliations:** 1 Department of Chemistry, Saint Louis University, St. Louis, MO USA; 2 Department of Biochemistry, University of Missouri, Columbia, MO USA; 3 Bond Life Sciences Center, University of Missouri, Columbia, MO USA; 4 Department of Physics, University of Missouri, Columbia, MO USA; 5 Department of Chemistry, Indiana University, Bloomington, IN USA; 6 Institute for Data Science and Informatics, University of Missouri, Columbia, MO USA; 7 Department of Biological Engineering, University of Missouri, Columbia, MO USA; 8 Department of Molecular Microbiology and Immunology, University of Missouri, Columbia, MO USA; 9 Present Address: KCAS, LLC, Shawnee, KS USA; 10 Present Address: Pfizer, Biomedicine Design, Cambridge, MA USA; 11 Present Address: Laronde, Inc., Cambridge, MA USA; 12 Present Address: GRAIL, Menlo Park, CA USA

**Keywords:** NMR spectroscopy, RNA, Riboswitches

## Abstract

The discovery of ribozymes has inspired exploration of RNA’s potential to serve as primordial catalysts in a hypothesized RNA world. Modern oxidoreductase enzymes employ differential binding between reduced and oxidized forms of redox cofactors to alter cofactor reduction potential and enhance the enzyme’s catalytic capabilities. The utility of differential affinity has been underexplored as a chemical strategy for RNA. Here we show an RNA aptamer that preferentially binds oxidized forms of flavin over reduced forms and markedly shifts flavin reduction potential by −40 mV, similar to shifts for oxidoreductases. Nuclear magnetic resonance structural analysis revealed π–π and donor atom–π interactions between the aptamer and flavin that cause unfavorable contacts with the electron-rich reduced form, suggesting a mechanism by which the local environment of the RNA-binding pocket drives the observed shift in cofactor reduction potential. It seems likely that primordial RNAs could have used similar strategies in RNA world metabolisms.

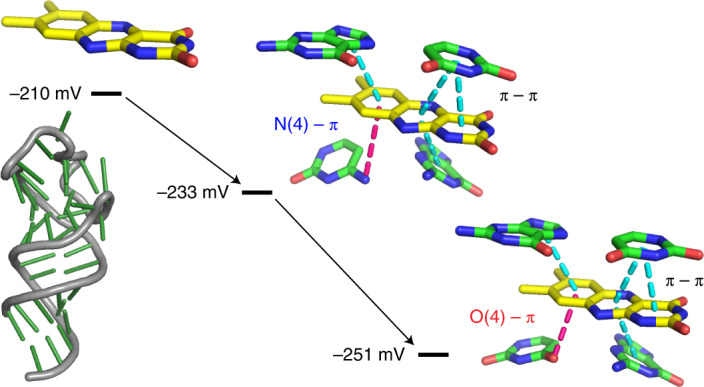

## Abstract (web summary)

An RNA aptamer that selectively binds FAD over FADH_2_ shifted the reduction potential of the bound cofactor, similar to flavoproteins, and was shown through structural characterization to use π–π and donor–π interactions to drive the shift.

## Main

The discovery of natural RNA enzymes (ribozymes)^1,2^ and their presence in all three domains of life^2,3^ has inspired researchers to explore the potential of RNA molecules to serve as primordial catalysts. A hypothesized RNA world^4^ could have made extensive use of diverse RNAs to support replication and metabolism. Natural ribozymes carry out RNA cleavage, ligation and transesterification reactions^1^, and the ribosome catalyzes peptide bond formation^5^. In vitro selection experiments^6,7^ have expanded the chemical repertoire of RNA to include nucleotide synthesis^8^, RNA polymerization^9^, carbon–carbon bond formation^10^ and several other reactions. For modern protein enzymes, the chemical environments of substrate-binding pockets are powerful determinants of enzyme reactivity. RNA has a remarkable ability to form binding pockets for potential substrates or cofactors, as seen in the aptamer portions of riboswitches and in artificial aptamers selected in vitro^11,12^. In a few cases, structural and mechanistic studies have provided examples of how local RNA environments can influence the intrinsic activity of bound molecules. For example, fluorogenic aptamers demonstrate how global RNA folding and local environments can enhance intrinsic fluorescence by desolvating the ligand and constraining rotation of the excited state^13^, and several ribozymes provide examples of RNA environments that shift pK_a_ values of bound ligands^14–16^. It seems likely that RNA binding pockets can employ additional mechanisms to influence ligand properties for catalytic use, such as by leveraging thermodynamic properties in ways proteins do through ground state perturbation and differential affinities for substrate and product.

**Fig. 1:**
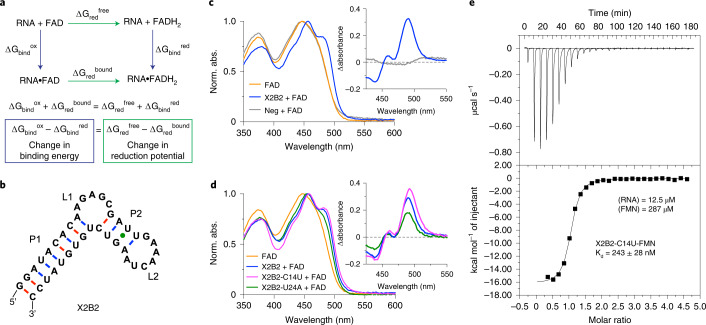
Aptamer X2B2 binding leads to red-shifted flavin absorbance and new peaks. **a**, Relationships among Gibbs free energies of binding and reduction. Diagram shows how differential binding between oxidized and reduced forms of the flavin cofactor such as FAD leads to a change in E_m_. **b**, Secondary structure of aptamer X2B2 as initially predicted by mfold^43^. This structure served as the basis for mutational analysis. **c**, UV-Vis spectra of free FAD (orange), X2B2-FAD complex (blue) and a non-FAD-binding RNA with added FAD (Neg; gray), showing the spectral changes that occur upon FAD binding to RNA. Inset shows the difference in absorbance between FAD that is bound to X2B2 and free FAD to highlight the changes in the spectra. **d**, UV-Vis spectra of free FAD (orange), FAD bound with X2B2 (blue), X2B2-C14U (magenta) and X2B2-U24A (green). Inset shows difference in spectrum relative to free FAD. **e**, Representative ITC thermogram of titrating FMN into X2B2-C14U in the top panel along with peak integration to determine K_d_ in the bottom panel. K_d_ value shown is the mean ± s.d. of *n* = 3 independent experiments.

Redox reactions are foundational for modern metabolism, both for biosynthesis and catabolism. Adenosine monophosphate-containing cofactors, such as nicotinamide adenine dinucleotide (NAD^+^) and flavin adenine dinucleotide (FAD), are often used by key enzymes in metabolic pathways. They are often viewed as strong candidates for having been part of an early RNA world^17^, and several studies have suggested that flavin-type molecules could have formed under plausible prebiotic conditions^18^. Nevertheless, RNA’s ability to exploit these cofactors for redox catalysis remains underexplored. A cardinal rule of redox metabolism is that electrons flow down the energy gradient, which is defined by the midpoint reduction potentials (E_m_) of electron donor and acceptor species. Free flavins have E_m_ values around −210 mV and, hence, can only transfer electrons to substrates with E_m_ values that are more positive than this value and can only receive electrons from substrates with E_m_ values that are more negative. Protein enzymes markedly shift the E_m_ values of bound flavins by exploiting the energy of differential recognition of oxidized and reduced cofactors (Fig. 1a), enabling them to react with substrates that span a broader range of E_m_ values. Flavins non-covalently bound within flavoenzymes have E_m_ ranging from 0 mV to −360 mV, whereas the E_m_ of covalently bound flavins can be shifted as far as +160 mV (ref. ^19^). This wide range of observed E_m_ among flavoenzymes illustrates the tremendous power of local chemical environments within protein-binding pockets to expand the reactive capabilities of flavins so that they can participate in diverse reactions, such as the oxidation of succinate to fumarate^20^ or the dehalogenation of chlorophenols^21^. If RNA can similarly leverage differential recognition of oxidized and reduced cofactors to shift E_m_ values, this would provide a mechanism by which primordial RNA could have catalyzed diverse metabolic reactions in an RNA world, in addition to providing a starting point for engineering diverse oxidoreductase ribozymes for synthetic biology applications.

Previous in vitro selection experiments have identified aptamers that recognize nicotinamides and flavins, but preferential binding to the oxidized over the reduced forms was not enforced during the selections. From those previous efforts, a preference for oxidation state was reported only for an NAD^+^ aptamer^22,23^, and changes to the E_m_ of the bound NAD^+^ were not reported. DNA aptamers that bind to the redox cofactor pyrroloquinoline quinone (PQQ) were found to leave the E_m_ of bound PQQ unaltered in cyclic voltammetry experiments, whereas DNA aptamers binding to the adenosine of NAD^+^ also had no impact on E_m_ (ref. ^24^). In vitro selection experiments identified an NAD^+^-dependent alcohol dehydrogenase ribozyme that uses NAD^+^ to oxidize a benzylic alcohol to an aldehyde and performs the reverse reaction with the reduced form, NADH^25–27^. This ribozyme possesses the ability to alter the rate of the oxidation and reduction reactions, a kinetic property, but changes to the E_m_ of the cofactor, a thermodynamic property, were not reported. Ribozymes that use flavin redox cofactors, such as FAD, have not been reported. Our previous work provided an early example of an RNA that recognizes the FAD isoalloxazine ring, but the aptamer from that study, Ftest1, did not differentiate between oxidized and reduced flavins^28^. These examples demonstrate that differential binding to cofactors is not a generic property for nucleic acids and that appropriate binding pockets capable of impacting cofactor properties do not automatically emerge during selection experiments. In contrast, we report in this study an RNA aptamer that preferentially binds oxidized flavins and markedly shifts their E_m_ values. Nuclear magnetic resonance (NMR) structural studies provide key insights that reveal the mechanisms by which the local chemical environments within the RNA binding pocket achieve these shifts.

## Results

### Aptamers specific for oxidized flavin

**Extended Data Fig. 1:**
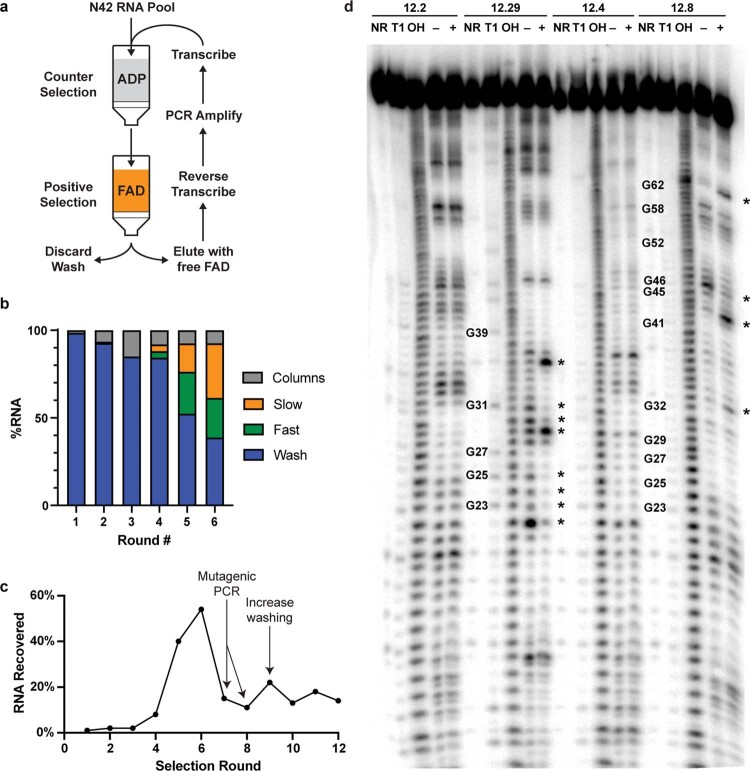
Overview and results of in vitro selections. **a**, In vitro selection design and (**b**) elution profile for rounds 1–6 of the selection showing percentage of RNA that was washed off (blue), eluted in ‘fast’ elution (green), eluted in ‘slow’ elution (orange), and remained on the ADP or FAD column (grey) after elutions were completed. **c**, Percent of RNA recovered (slow and fast elution in rounds 1–6 and only slow elution from rounds 7–12) at each round of *in vitro* selection. Mutagenic PCR was performed during amplification steps following rounds 7 and 8, and an increase in washing volume started at round 9 and was continued through round 12 (ref.^49^). **d**, Gel image showing in-line probing cleavage pattern of different RNA aptamers in absence (–) and presence (+) of 500 µM FAD. Unreacted RNA, T1 ladder and alkaline digestion ladders are indicated by NR, T1 and OH, respectively. Asterisks represent significant changes in cleavage patterns between the two conditions. A single trial for each aptamer was carried out for 18 h at 37 °C in 25 mM Tris pH 8.0 and 30 mM MgCl_2_ (ref. ^51^). Source data

**Extended Data Fig. 2:**
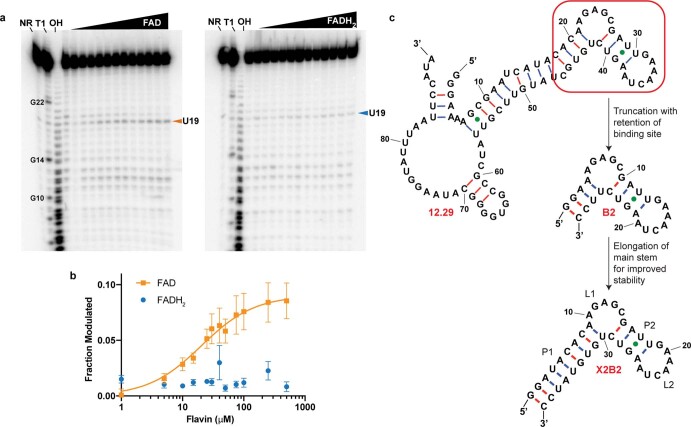
Binding selectivity towards FAD over FADH_2_ and aptamer minimization. **a**, Gel images showing in-line probing patterns of aptamer B2 at 0, 1, 5, 10, 15, 25, 30, 40, 50, 75, 100, 250 or 500 µM FAD (left) or FADH_2_ (right). Unreacted RNA, T1 ladder and alkaline digestion ladders are indicated by NR, T1 and OH, respectively. Reactions were carried out for 12 h at 37 °C in 25 mM Tris pH 8.0 and 30 mM MgCl_2_. At least four independent experiments produced similar results. **b**, Cleavage signals at U19 of B2 were normalized to unreacted RNA (full length) to calculate fraction modulated at each concentration of FAD. Error bars indicate standard error of the mean from at least four independent experiments. Curve fits to one-site binding with shared B_max_ gave an apparent dissociation constant of approximately 20 µM for FAD binding to aptamer B2, while the dissociation constant for FADH_2_ could not be accurately determined^51^. **c**, Aptamer 12.29 was truncated to a 28-nucleotide active aptamer, named B2. The main stem of aptamer B2 was extended for possible improved stability to yield aptamer X2B2, which was then used in UV-Vis analysis, mutational survey, redox assays, NMR spectroscopy and ITC. These preliminary secondary structures were predicted by mfold^43^. Source data

In vitro selection identified RNA aptamers with affinity for FAD (Extended Data Fig. 1 and Supplementary Table 1). Although aptamers 12.8 and 12.29 showed FAD-induced changes in their in-line probing cleavage patterns, aptamer B2 (a truncated version of aptamer 12.29) was unaffected by FADH_2_ (Extended Data Fig. 2a,b), suggesting that it preferentially binds to the oxidized form. Extending the main stem of B2 increased its overall stability and yielded a 38-nucleotide aptamer named X2B2 (Fig. 1b and Extended Data Fig. 2c) that was studied further in this work.

**Extended Data Fig. 3:**
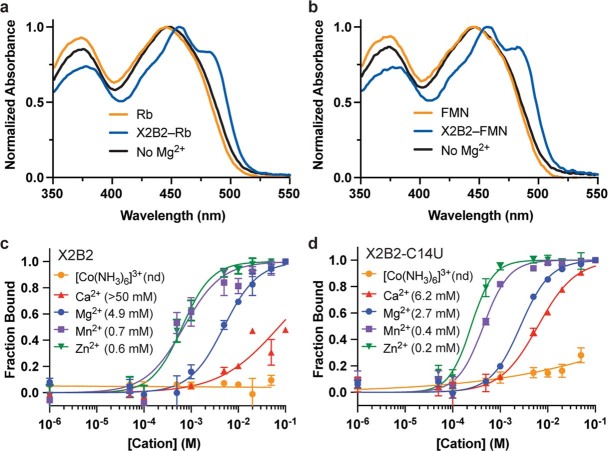
Binding condition characterizations of X2B2 and X2B2-C14U. X2B2 showed the same UV-Vis shift characteristics for both (**a**) riboflavin and (**b**) FMN. For all flavins, Mg^2+^ was needed for binding. Fluorescent quenching of FMN was used to determine binding with Mg^2+^ (blue), Mn^2+^ (purple), Ca^2+^ (red), Zn^2+^ (green) or [Co(NH_3_)_6_]^3+^ (orange) for (**c**) X2B2 and (**d**) X2B2-C14U. For each condition, the indicated metal ion was provided as the only divalent ion, at concentrations of 0.001, 0.05, 0.1, 0.5, 1, 5, 10, 20, 50 and 100 mM. For both aptamers, Mg^2+^, Mn^2+^ and Zn^2+^ facilitated full binding capability, while for Ca^2+^, only X2B2-C14U retained full binding ability whereas X2B2 was not fully binding. For both aptamers, [Co(NH_3_)_6_]^3+^ did not facilitate binding, indicating that at least one partially or fully dehydrated magnesium is crucial for binding. Values of [M^2+^]_1/2_ (divalent metal ion concentration at which half-maximal fraction bound was observed) are shown in parathesis in figure legends for both X2B2 and X2B2-C14U. Data are presented as mean±s.d. for n = 3 independent experiments.

RNA–flavin complex formation was readily monitored by ultraviolet-visible (UV-Vis) spectroscopy. Free FAD absorbs maximally (λ_max_) at 450 nm with a smaller second peak at 377 nm. In the presence of aptamer X2B2, λ_max_ of the bound FAD was red-shifted to 456 nm (Δλ_max_ = +8 nm), with shoulders at 482 nm and 430 nm and a second peak at 384 nm (Fig. 1c). Denaturing conditions (urea, heat or EDTA) or replacing X2B2 with an unrelated, non-flavin-binding control RNA returned the absorbance peaks to that of free FAD, indicating that the spectral shifts were due to aptamer binding. Identical peak shifts were observed for X2B2 binding to riboflavin (Rb) and flavin mononucleotide (FMN) (Extended Data Fig. 3a,b), indicating that the primary recognition moiety was the flavin isoalloxazine ring. Although Mg^2+^ was used as the divalent metal ion during the selection, flavin binding by aptamer X2B2 was also observed when Mg^2+^ was replaced with Mn^2+^, Zn^2+^ or (to a lesser degree) Ca^2+^ (Extended Data Fig. 3c), similar to the permissive metal ion requirements of other flavin-binding RNAs^29,30^. Cobalt(III) hexammine, which mimics hydrated Mg^2+^ and for which the NH_3_ ligands are exchange inert, did not support FAD binding, indicating at least one required inner-sphere contact with a partially dehydrated metal ion (Extended Data Fig. 3c).

**Extended Data Fig. 4:**
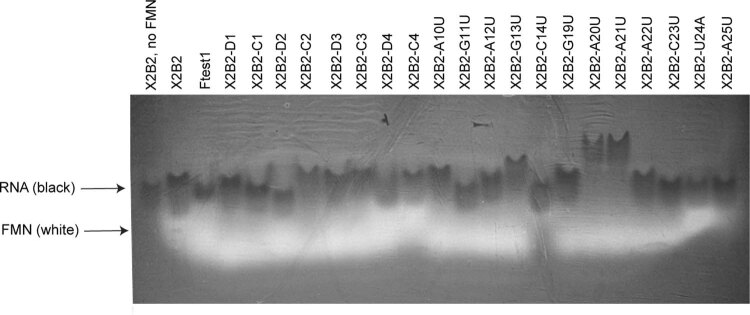
Native PAGE analysis of X2B2 and mutants. Aptamer X2B2 (with and without FMN) was analyzed using native PAGE alongside Ftest1 and several X2B2 mutants with FMN. Image shown is an overlay of images taken using shortwave UV (254 nm, UV shadow of nucleic acid, black) and longwave UV (365 nm, fluorescence of FMN, white). Note for X2B2-C14U the fluorescence of FMN is quenched due to aptamer binding. Experiments were independently repeated three times with similar results. Source data

**Extended Data Fig. 5:**
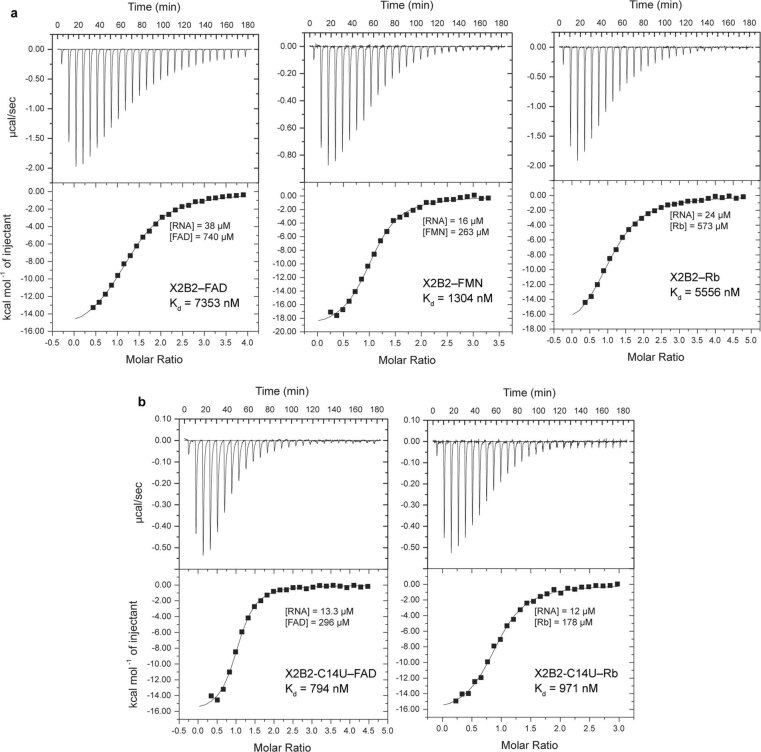
ITC thermograms of titrations of flavins into RNA aptamers. **a**, Representative ITC thermograms for X2B2 titrated with FAD (left), FMN (center), and riboflavin (right). Starting concentrations of X2B2 and flavin are shown along with the calculated K_d_ value for each. **b**, Representative ITC thermograms for X2B2-C14U titrated with FAD (left) and riboflavin (right). Starting concentrations of X2B2-C14U and flavins are shown along with the calculated K_d_ value for each. The statistics of thermodynamic parameters were calculated from n = 3 independent experiments and summarized in Supplementary Table 3.

To better understand the RNA nucleotide and structural requirements of X2B2 for flavin binding, disrupting and compensatory rescue mutations were made within the initially predicted base-paired stems (P1 and P2; Fig. 1b), and nucleotides in the predicted loop regions (L1 and L2; Fig. 1b) were changed. Mutations in P1 did not significantly alter FAD binding (Supplementary Table 2). Although most mutations in P2, L1 and L2 were highly disruptive to both flavin-binding ability (Supplementary Table 2) and overall RNA structure (Extended Data Fig. 4), two single-nucleotide mutants in each loop region retained binding. The UV-Vis spectrum of one of these mutants, X2B2-C14U, showed peaks that were more strongly red-shifted (λ_max_ = 458 nm and Δλ_max_ = +10 nm) than for X2B2 itself (Fig. 1d). The X2B2-C14U mutant also displayed the same divalent metal ion dependence as the parent aptamer and was able to bind FAD with the use of Mn^2+^, Ca^2+^ and Zn^2+^. Binding was not supported by the use of cobalt(III) hexammine, indicating the presence of at least one inner-sphere contact interaction in the mutant (Extended Data Fig. 3d). Finally, thermodynamic parameters of X2B2 and X2B2-C14U interacting with FAD, FMN and Rb were determined by isothermal titration calorimetry (ITC) (Extended Data Fig. 5 and Supplementary Table 3). All the tested binding events were exothermic with micromolar to nanomolar affinities and the highest affinity (K_d_ = 243 ± 28 nM) observed for X2B2-C14U with FMN (Fig. 1e), further supporting that ligand recognition is primarily through the isoalloxazine ring.

### Aptamer binding shifts flavin E_m_

**Fig. 2:**
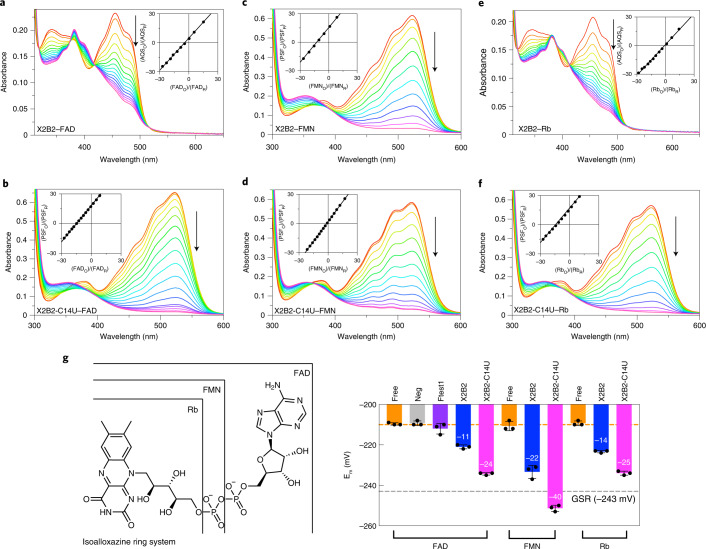
Aptamers X2B2 and X2B2-C14U shift the midpoint reduction potential of bound flavins. UV-Vis spectra of the redox assay for X2B2–FAD and AQS (**a**), X2B2-C14U-FAD and PSF (**b**), X2B2–FMN and AQS (**c**), X2B2-C14U–FMN and PSF (**d**), X2B2–Rb and AQS (**e**) and X2B2-C14U–Rb and PSF (**f**). Insets are the linear best fits used to calculate E_m_, which are given by the displacement of the *y*-axis intercept relative to the reference dye (AQS = −225 mV; PSF = −252 mV), as detailed in Extended Data Fig. 6d. Axes shown represent the values of 12.5ln(oxidized/reduced). Arrows and the gradient of line colors indicate direction of spectral change with time. **g**, Structures of FAD, FMN and Rb (left) and E_m_ of individual flavins and aptamer–flavin complexes (right). Numbers within the bars depict ΔE_m_ (the change in E_m_ from those of the free flavins). Bars without numbers indicate no significant change in E_m_. The orange dashed line represents the average E_m_ of all three flavins (−210 mV). For comparison, the gray dashed line shows the E_m_ of the flavoprotein GSR (glutathione reductase)^44^. Data are presented as mean ± s.d. for *n* = 3 independent sets of measurements.

**Extended Data Fig. 6:**
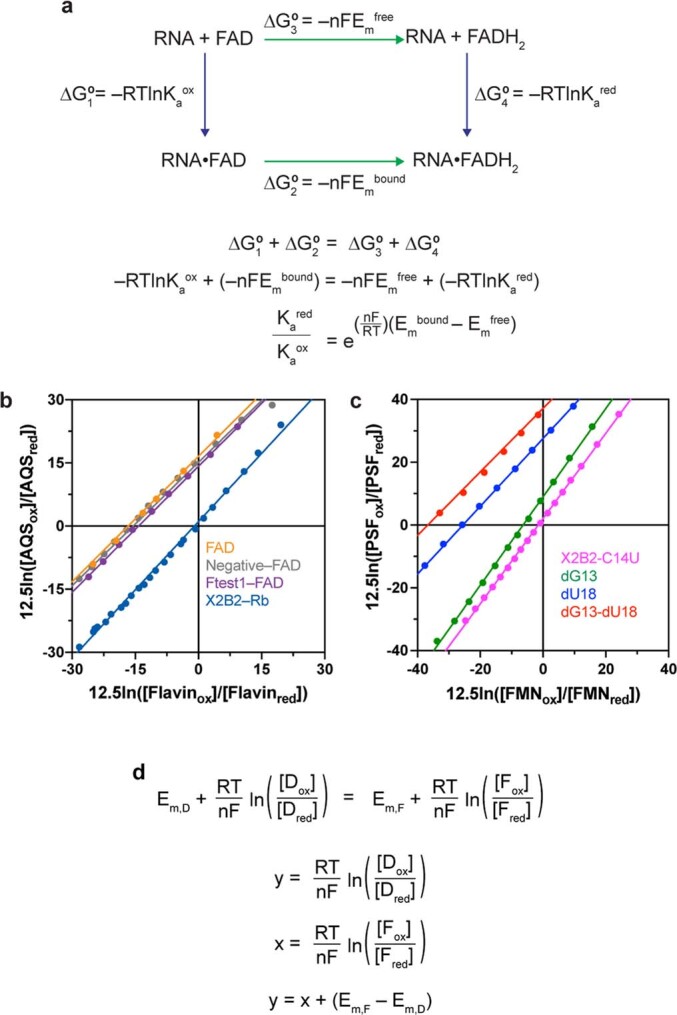
Equations for differential binding constants and additional redox assays. **a**, Diagram from Fig. 1a with formulas relating ΔG° values to binding constants (K) or to midpoint potentials (E_m_). F represents the Faraday constant. The two thermodynamic pathways are set equal to each other and rearranged to get the theoretical relationship between the fold difference in binding constants (K_a_^red^/K_a_^ox^) and the change in reduction potential (E_m_^bound^ – E_m_^free^). **b**, Redox assay plots for FAD, Neg-FAD, Ftest1-FAD and X2B2-Rb. **c**, Redox assay plots comparing X2B2-C14U along with the deoxyribose mutants dG13, dU18 and dG13-dU18. **d**, Derivation of the linear response of the redox plots. For these plots, the natural log (ln) of the ratio of oxidized:reduced dye is plotted against the natural log (ln) of the ratio of oxidized:reduced flavin at each time point (both multiplied by 12.5 to account for the nF/RT term from the Nernst equation, where n = 2). In the line of best fit for each data set, the *y*-intercept (b) equals the difference in reduction potential between the flavin and the reference dye (b = E_m,F_ – E_m,D_) in millivolts.

To determine whether aptamer binding to the flavin shifts its reduction potential, E_m_ of the bound flavin was measured using a xanthine oxidase-coupled enzyme assay that simultaneously monitors oxidized-to-reduced ratios for the flavin and a reference dye^31,32^. Using anthraquinone-2-sulfonic acid (AQS, E_m_ = −225 mV) as the reference dye, E_m_ values for free FAD, FMN and Rb were −209 ± 1 mV, −211 ± 2 mV and −210 ± 1 mV, respectively, which agreed with literature values^33^. A non-FAD-binding RNA (Neg) was tested with FAD, and an E_m_ of −209 ± 1 mV was measured, showing that non-FAD-binding RNA does not affect the E_m_ of the flavin. Additionally, Ftest1, an RNA aptamer that does not distinguish between the oxidized and reduced forms of FAD^28^, was tested with FAD, and an E_m_ of −212 ± 3 mV was measured, showing that non-preferential binding to one form of FAD toward the other does not substantially alter E_m_ (Extended Data Fig. 6b). In contrast, aptamer X2B2 (Fig. 2a) shifted E_m_ to −223 ± 1 mV, representing ΔE_m_ of −11 mV relative to free FAD, and X2B2-C14U, for which phenosafranin (PSF, E_m_ = −252 mV) was used as the reference dye, shifted E_m_ to −234 ± 1 mV, which was a ΔE_m_ of −25 mV versus free FAD (Fig. 2b).

If the observed E_m_ shifts observed for FAD are driven by RNA–flavin interactions, then they should also be observed for aptamer complexes with FMN and Rb. Indeed, the X2B2–FMN complex had E_m_ of −234 ± 3 mV (ΔE_m_ = −23 mV; Fig. 2c), and the X2B2-C14U–FMN complex had the most negative E_m_ value observed in this study at −251 ± 2 mV (ΔE_m_ = −40 mV; Fig. 2d). The X2B2–Rb complex had E_m_ of −223 ± 1 mV (ΔE_m_ = −14 mV; Fig. 2e), and the X2B2-C14U–Rb complex had E_m_ of −234 ± 1 mV (ΔE_m_ = −25 mV; Fig. 2f). The results of the redox assays along with the structures of the different flavins tested are summarized in Fig. 2g.

### NMR analysis of X2B2-C14U–FMN complex

**Fig. 3:**
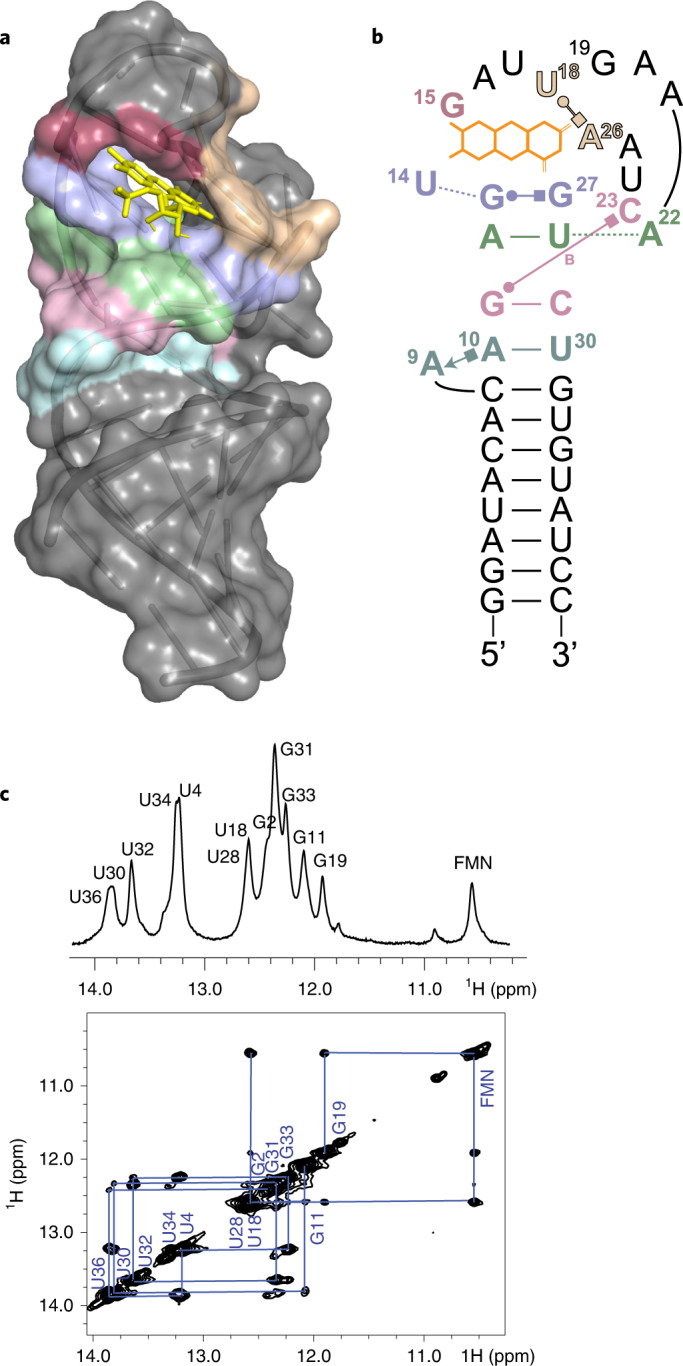
Structure of the X2B2-C14U–FMN complex. **a**, 3D representation of the X2B2-C14U–FMN complex with the RNA shown in cartoon and surface view and FMN in stick view. Colored surface areas represent noteworthy nucleobases that are shown in subsequent panels and in Figs. 4 and 5 using the same color scheme. **b**, Experimentally determined secondary structure of X2B2-C14U. The bound flavin is shown as three orange rings. Watson–Crick base pairs are shown with dashes, and non-canonical hydrogen bonds are shown with Leontis–Westhof annotation (B, bifurcated base pair)^45^. **c**, Imino proton assignments for the X2B2-C14U–FMN complex with the 1D (top) and 2D (bottom) spectra.

**Fig. 4:**
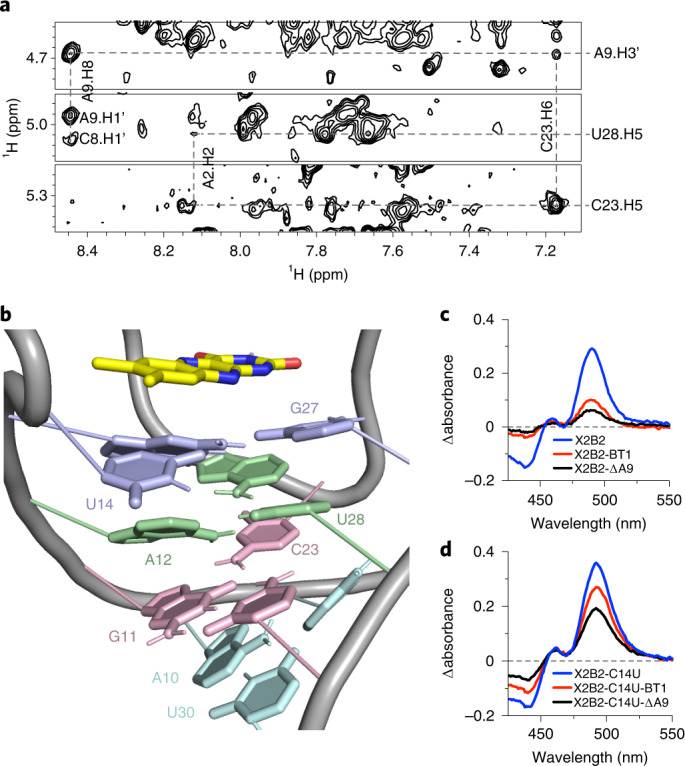
Structural study of the X2B2-C14U–FMN complex reveals an extended base-triple platform under the bound flavin. **a**, Portions of the 2D NOESY spectra for X2B2-C14U–FMN show NOEs of C23 with A9 and A22, suggesting that A22 and C23 fold back to stack on A9 and form a base-triple platform. **b**, Base-triple platform, with nucleotides color-coded by triples in cyan (U30:A10•A9), pink (C29:G11•C23), green (A12:U28•A22) and purple (U14•G13•G27). **c**,**d**, UV-Vis spectra showing the difference in absorbance between bound and free FAD for the base-triple mutants (BT1, shifted U30:A10•A9 base triple; ΔA9, removal of A9), as tested for X2B2 (**c**) and X2B2-C14U (**d**).

To define the possible modes of interactions within the aptamer–flavin complex, we turned to structural characterization by NMR spectroscopy. The X2B2-C14U–FMN complex was chosen for structural characterization, as it gave the highest affinity by ITC and the largest ΔE_m_ in the xanthine oxidase redox assays. Using molecular dynamic simulations with NMR-derived distance restraints, a structure was determined (Fig. 3a,b) that was distinct from the originally predicted secondary structure. Assignment of imino protons in X2B2-C14U–FMN confirmed the predicted bottom stem involving nucleotides 1–8 and 31–38 (Fig. 3c). Two-dimensional (2D) NOESY showed nuclear Overhauser effecs (NOEs) between the main stem and loop nucleotides A22 and C23, suggesting that the apical loop residues fold back and insert into the major groove (Fig. 4a). This loop motif is part of a multi-layer base-triple platform that builds up the main stem to the *si*-face of the bound flavin (Fig. 4b). Because the U30:A10•A9 base triple was several nucleotides below the flavin-binding pocket, mutations were introduced to determine the importance of this base triple in both X2B2 and X2B2-C14U. These mutations either removed A9 and left just the U30:A10 base pair (X2B2-ΔA9 and X2B2-C14U-ΔA9) or relocated the entire base triple down the stem by two base pairs (X2B2-BT1 and X2B2-C14U-BT1), thus moving it further away from the flavin-binding pocket. Both changes in X2B2 caused almost complete loss of flavin binding (Fig. 4c), demonstrating that this base triple is particularly important for flavin binding despite being far from the flavin-binding site, potentially by anchoring formation and stabilization of the other three base triples in the platform. In X2B2-C14U, which forms a more stable complex with FMN than does X2B2, both changes caused a decrease, but not a complete loss, in binding ability, with ΔA9 having a greater effect than the shifted base triple (Fig. 4d).

**Fig. 5:**
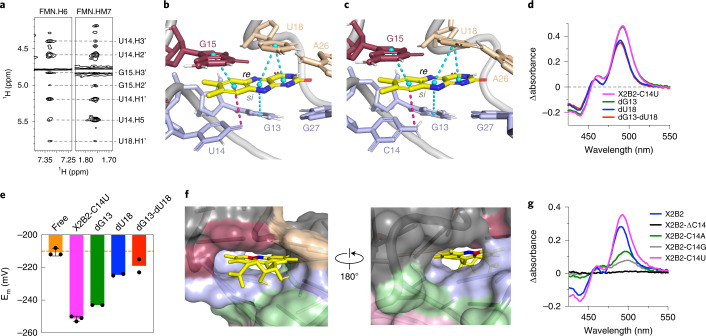
The flavin-binding pocket consists of π–π and donor atom–π interactions. **a**, Portions of the 2D NOESY spectra of the X2B2-C14U–FMN complex show intermolecular NOEs, suggesting that the xylene ring of the FMN is sandwiched between U14 and G15. **b**, Flavin-binding pocket for X2B2-C14U, showing π–π stacking (cyan) and donor atom–π (pink) interactions between FMN and nearby nucleotides, where distances less than or equal to 4.5 Å are shown as dashed lines. **c**, Same interactions and distances for X2B2. **d**, UV-Vis spectra showing the difference in absorbance between free and bound FMN for X2B2-C14U (magenta) and its mutants with deoxyribose sugars at position G13 (green, under magenta), U18 (blue) or both (red, under blue). **e**, E_m_ values for FMN (−211 mV), X2B2-C14U (−251 mV) and its deoxyribose mutants, dG13 (−243 mV), dU18 (−225 mV) and dG13-dU18 (−219 mV). For FMN and X2B2-C14U, data are presented as mean ± s.d. for *n* = 3 sets of independent measurements. For the deoxyribose mutants, only two measurements were taken owing to limited sample availability. **f**, Surface views of the FMN-binding pocket, showing that the reactive C4a (red) and N5 (blue) atoms are solvent accessible. **g**, UV-Vis spectra showing the difference in absorbance between bound and free FAD for the mutants at nucleotide position 14.

The uracil edge of the bound FMN pyrimidine ring formed neither a Watson–Crick nor a Hoögsteen base pair with an aptamer adenosine, as evidenced by the chemical shift of FMN HN3 (Supplementary Fig. 1). Additionally, the NOE between FMN HN3 and G19 was much weaker than the NOE observed in a typical G•U wobble, suggesting that FMN was not paired with G19 (Fig. 3c and Supplementary Fig. 1a). In the binding pocket, NOEs were detected between H6 and H7α (methyl hydrogens) of FMN with U14 and G15 (Fig. 5a and Supplementary Fig. 1b,c), suggesting that the FMN xylene ring is sandwiched between U14 and G15 via π–π stacking, with U14 interacting with the FMN *si-*face and G15 interacting with the *re*-face (Fig. 5b). NMR data were also collected for X2B2–FMN, for which the NOE patterns were almost identical to that of X2B2-C14U–FMN (Supplementary Fig. 2), demonstrating that X2B2 and X2B2-C14U share the same flavin-binding mode (Fig. 5c).

A few of the structures in the molecular dynamics (MD) ensemble placed the 2′-OH of either G13 or U18 within 3.3 Å of the N5 of FMN, indicating a possible source of hydrogen bonding between the RNA and flavin. Mutant X2B2-C14U with deoxy substitutions at one or both of these positions were tested for their ability to bind to FMN. Each variant still produced a red-shift in the UV-Vis spectra, with a modest loss of the shoulder at 482 nm when the deoxy substitution was included at U18 (Fig. 5d). The impact on E_m_ was also tested, and the deoxy substitution at G13 had E_m_ = −243 mV (ΔE_m_ = −32 mV); the deoxy substitution at U18 had E_m_ = −225 mV (ΔE_m_ = −14 mV); and the deoxy substitution at both G13 and U18 had E_m_ = −223 mV (ΔE_m_ = −12 mV) (Fig. 5e and Extended Data Fig. 6c). These shifts are less than those observed for the all-ribose version of the aptamer.

The resulting model (Fig. 5f) indicated that the C4a and N5 of FMN are solvent accessible, with the isoalloxazine ring having a solvent-accessible surface area of 7.1 Å^2^ (compared to 336.7 Å^2^ for unbound isoalloxazine ring), which allows the FMN to react with other molecules, such as the methyl viologen mediator used in the xanthine oxidase assay, and potentially with substrates in engineered oxidoreductase ribozymes.

Because of the proximity of the nucleotide at position 14 to the bound FMN and the powerful impact of its identity (C or U) on affinity and ΔE_m_, we introduced a purine (X2B2-C14A and X2B2-C14G) or deletion (X2B2-ΔC14) at that position. From the UV-Vis spectra, X2B2-ΔC14 lost all flavin-binding ability, and the purine mutants retained only a minor amount of binding (Fig. 5g and Supplementary Table 2), highlighting the importance of a pyrimidine in position 14 for flavin binding.

## Discussion

Using in vitro selection, we isolated an RNA aptamer with preferential binding to FAD over FADH_2_ and successfully minimized the aptamer to a length of 38 nucleotides, resulting in aptamer X2B2. Using UV-Vis spectroscopy, we observed aptamer-induced changes to the flavin spectra, specifically red-shifted peaks and new shoulders. These changes facilitated analysis of aptamer mutants and allowed us to identify a key mutant, X2B2-C14U, with greater binding affinity for the flavin. Red-shifts of similar magnitude have been observed in some flavoproteins, where they are attributed to protein–flavin interactions^34^, and the appearance of shoulders at ±26 nm relative to λ_max_ indicates a lack of hydrogen bonding with the flavin relative to the free flavin in water^35–37^. Both of these aptamers readily bound to FAD, FMN and Rb, which suggested that the interactions between the aptamers and the cofactors were concentrated on the isoalloxazine ring, and this binding was maintained under a wide range of divalent metal ion conditions.

Via an enzymatic assay, we measured a ΔE_m_ for aptamer-bound flavin relative to free flavin, demonstrating, to our knowledge for the first time, that an RNA is capable of shifting the E_m_ of a redox cofactor. Our observed ΔE_m_ values corresponded to a greater than twofold increase in K_d_^red^ relative to K_d_^ox^ for X2B2 and a nearly sevenfold increase for X2B2-C14U, indicating that X2B2-C14U was better able than the parent X2B2 aptamer to distinguish between FAD and FADH_2_ (see Extended Data Fig. 6a for equation and Supplementary Table 4 for calculated ratios). When considering the measured E_m_ for FMN bound to X2B2-C14U, the value is similar to that observed in some flavoenzymes such as glutathione reductase^38^ (dashed line in Fig. 2g) and corresponds to a calculated 23-fold difference between K_d_^red^ and K_d_^ox^. Both aptamers achieved a larger ΔE_m_ for FMN than for FAD, indicating that the adenosine moiety of FAD negatively impacts binding to these aptamers. Intramolecular π–π stacking of the adenine nucleobase with the isoalloxazine ring in free flavin may require that unstacking occurs before binding, thereby presenting an energetic cost. The adenosine could also have steric clashes with the aptamer or interact with aptamer nucleobases and disrupt flavin positioning within the complex.

By comparing the ITC results and the E_m_ values with the different flavins, we sought to identify possible trends in the data related to ligand binding. It must be noted that these assays have different relationships to the binding interactions. The ΔE_m_ is related to the differential binding of the oxidized form relative to the reduced form of the flavin ring, with little or no contribution from interactions with other parts that may be present on the cofactor. In contrast, the binding constants determined by ITC encompass the binding interactions of the entire cofactor, which can include contacts and/or clashes not only with the flavin ring but also with other components of the cofactor. So, a direct trend is not inevitable as the ribitol chain, phosphate and/or adenosine in the different flavin cofactors may impact the binding constants without impacting the differential binding of oxidized versus reduced form. When comparing the two aptamers, we consistently observed tighter binding and larger ΔE_m_ for X2B2-C14U compared to X2B2, regardless of the flavin used. For the different flavins, both aptamers displayed the strongest binding and the largest ΔE_m_ values with FMN. Despite the difference between the simple ribitol chain (Rb) and the ribitol plus ADP (FAD) attached to the isoalloxazine ring, these two flavins bound with similar affinity to each aptamer and produced similar, more modest ΔE_m_ values relative to FMN. Nevertheless, all six complexes (two aptamers × three flavins) substantially shifted E_m_ for the bound flavin cofactors, and X2B2-C14U was especially considerable in decreasing E_m_ values by as much as ΔE_m_ = −40 mV (Fig. 2g).

The NMR-derived structure of X2B2-C14U bound to FMN provides a picture of the flavin-binding pocket and how the aptamer is able to impact the reduction potential of flavin. Flavin contains a pyrimidine ring with a uracil face that could form a base pair with adenosine, as has been observed in an unrelated aptamer identified by Burgstaller and Famulok^22,39^ and in the FMN riboswitch^30^. In stark contrast, X2B2-C14U does not hydrogen-bond to this uracil face of FMN. The lack of hydrogen bonding to the FMN pyrimidine ring in X2B2-C14U is supported by the observed shoulders in our UV-Vis spectra (Fig. 1c,d)^35–37^. X2B2-C14U contains important base triples above and below the bound flavin, providing an environment for π–π stacking. The π–π stacking and lack of observed hydrogen bonds between the uracil edge of the FMN pyrimidine ring and RNA aptamer explain the observed overall negative E_m_ shifts of the aptamer–flavin complexes, based on similar observations from flavoproteins^40,41^. We also investigated potential hydrogen-bonding interactions between the N5 of FMN and the 2′-OH of G13 or U18. If these hydrogen bonds exist, removing them by substituting with deoxyribose sugars at G13 and/or U18 would lead to more negative ΔE_m_ as hydrogen-bonding interactions result in positive E_m_ shifts^40^. The deoxyribose mutants displayed the opposite result and, instead, had more positive E_m_. This indicates that the deoxyribose sugars at position G13 and/or U18 have a different conformation than their ribose counterparts, likely due to changes in the sugar pucker and resulting in the nucleobase being moved farther away from the bound flavin and reducing π–π stacking interactions. The effect on E_m_ for these deoxyribose mutants varied, with the deoxyribose U18 having a more positive E_m_ shift than the G13. This can potentially be attributed to the U18 uracil ring being closer to the bound flavin compared to the G13 guanine and having more influential π–π stacking interactions.

The identity of the pyrimidine at position 14 clearly influenced the E_m_ for the bound flavin. Nucleotide C14 in X2B2 interacts with the *si*-face of the xylene ring via the N4 exocyclic amine (electron withdrawing), whereas the U14 in X2B2-C14U interacts via the O4 atom (electron donating) (Fig. 5b,c). In comparing these donor atom–π interactions, the more electron-rich O4 has favorable interactions with the electron-deficient FMN, as indicated by a lower K_d_. Furthermore, the O4 atom has unfavorable interactions with the electron-rich FMNH_2_, which increases the ratio of K_d_^red^ to K_d_^ox^ and results in a more negative E_m_ (ref. ^42^). Thus, subtle changes within the binding pocket impact ΔE_m_ without disrupting the overall structure of the aptamer–FMN complex.

Our results demonstrate that the local chemical environment of an RNA-binding pocket is not merely a passive interaction surface but, instead, is capable of substantially shifting the midpoint reduction potential (E_m_) of a bound redox cofactor, flavin. Based on UV-Vis spectral changes and NMR structural analysis, the X2B2 aptamer and its X2B2-C14U mutant use interactions similar to those observed in flavoproteins, including π−π stacking and donor−π interactions with the isoalloxazine ring, to achieve the differential binding required for substantially shifting E_m_. Functional groups within the X2B2-binding pocket further modulate differential binding and the resulting ΔE_m_ values. Collectively, these results suggest catalytic strategies that early RNAs could have harnessed, providing them with a broadened range of cofactor reduction potentials, such as those now seen in flavoproteins and other oxidoreductase enzymes, and allowing them to develop metabolism in an RNA world to enable assembly of new functional RNAs with expanded catalytic abilities.

## Methods

### Reagents

DNA oligonucleotides were purchased from Integrated DNA Technologies (IDT) and were purified by denaturing PAGE. 9,10-anthraquinone-2-sulfonic acid sodium salt, hydrate (AQS) was purchased from Alfa Aesar. FAD, FMN and Rb were purchased from Chem-Impex. Xanthine, xanthine oxidase (grade IV, 4.5 mU µl^−1^), glucose oxidase (24.8 U mg^−1^), catalase (2,220 U mg^−1^), phenosafranin, methyl viologen and riboflavin-(dioxopyrimidine-^13^C_4_, ^15^N_2_) were purchased from Sigma-Aldrich, and hexaamminecobalt (III) chloride was purchased from Tokyo Chemical Industry (TCI). The FAD resin (Sigma-Aldrich) was agarose derivatized with cyanogen bromide–activated adipic dihydrazide. Radiolabeled α-^32^P GTP used for in vitro transcriptions during selections was purchased from ICN. For NMR spectroscopy studies, the nucleotide-specific ^2^H-labeled samples, including AC-, AG-, GU- and A^2R^U^R^CG-X2B2-C14U, were prepared by incorporating the corresponding deuterated and protonated NTPs in T7 transcriptions, as previously described^46^. Fully deuterated NTPs and H5, H6-deuterated CTP and UTP were purchased from Silantes and Cambridge Isotope Laboratories (CIL). H8-deuterated ATP (A^2R^) and GTP (G^R^) were prepared in-house^47^, and ^13^C/^15^N NTPs were purchased from CIL. For all buffers used, the pH is defined at room temperature unless otherwise stated. Spectral data analysis was done using Microsoft Excel 365 and GraphPad Prism 9.

### In vitro transcription

RNAs were generated via in vitro transcription^48^ using DNA templates. During the in vitro selection process, RNA pool sequences were labeled using α-^32^P GTP. RNA products were purified using denaturing PAGE, extracted from the gel using 300 mM NaOAc and ethanol precipitated before use in the next selection round^49^. For aptamers used in characterization studies, 1 µM primer (Supplementary Table 1) was annealed to 1 µM ‘bottom-strand’ DNA template (containing complement to primer and the desired RNA transcript) by denaturing at 95 °C for 3 minutes in 5 mM HEPES pH 7.5, 15 mM NaCl and 0.1 mM EDTA, followed by a 5-minute incubation on ice. Transcription was initiated by the addition of reaction mixture to a final concentration of 80 mM HEPES pH 7.5, 25 mM MgCl_2_, 3 mM of each NTP, 10 mM DTT, 2 mM spermidine and 3 μg of in-house-prepared T7 RNA polymerase per 100-μl reaction. Reactions proceeded at 37 °C for 4–7 hours and were quenched by the addition of EDTA pH 8.0 and NaCl to final concentrations of 64 mM and 168 mM, respectively. Three volumes of 100% ethanol was added, followed immediately by centrifugation at 16,000*g* for 1 minute. The supernatant was removed, and the pellet was dried in vacuo and reconstituted in water for desalting on a Sephadex G-50 gravity column. RNA oligonucleotides were eluted using TEN elution buffer (10 mM Tris pH 8.0, 300 mM NaCl and 1 mM EDTA) and ethanol precipitated before denaturing PAGE purification. Desired products were excised from the gel and extracted via the crush-and-soak method in TEN elution buffer, followed by ethanol precipitation. Recovered RNA oligonucleotides were quantified using a NanoDrop 2000c (Thermo Fisher Scientific). For RNAs used in ITC and NMR spectroscopy, in vitro transcriptions were carried out in 40 mM Tris-HCl pH 8.0, 5 mM DTT, 10 mM spermidine, 0.01% (v/v) Triton X-100, 20 mM MgCl_2_, 12 mM of each NTP (regular or isotopically labeled) and Ribolock RNase Inhibitor (80 U, Thermo Fisher Scientific). Transcription reactions were quenched with 1 M urea and 25 mM EDTA. RNAs were purified by denaturing PAGE, visualized by UV shadowing, electroeluted from the gel using elutrap (Whatman) and washed in Amicon ultra-centrifugal filters (10,000 MWCO). ^2^H- and ^13^C/^15^N-labeled RNAs were synthesized by incorporating corresponding ^2^H- or ^13^C/^15^N-labeled NTPs into in vitro transcriptions.

### In vitro selection, cloning and sequencing

Aptamers with specificity for FAD were selected on an affinity column with immobilized FAD^49^. The initial RNA pool for selection (Extended Data Fig. 1a) was transcribed using a 109-nucleotide DNA strand consisting of an N_42_ random region flanked by constant primer regions for PCR amplification and a phage T7 promoter for in vitro transcription. Pool and primer sequences are provided in Supplementary Table 1. To initiate each round, the radiolabeled RNA pool was heat denatured and refolded in 1× TKNCM buffer (50 mM Tris pH 7.0, 10 mM NaCl, 30 mM MgCl_2_, 140 mM KCl and 10 µM CaCl_2_) at room temperature and then passed through 200 µl of an ADP-modified agarose column to remove adenosine-binding RNAs and to enforce flavin binding (counter selection). The flow-through was passed through an FAD-modified agarose resin. Non-binding RNAs were washed off with 2 ml of 1× TKNCM buffer, and weak binders were ‘fast’ eluted with 1 ml of 5 mM FAD in 1× TKNCM buffer. ‘Slow elutions’ were done for high-affinity RNAs by incubating the column four times for extended periods (two 30-minute incubations, followed by two 1-hour incubations) with 300 µl of 5 mM FAD in 1× TKNCM buffer before collecting each flow-through. A final wash of 5 mM FAD in 1× TKNCM buffer was done after the fourth elution step. The ‘fast’ and ‘slow’ elutions and final wash were combined until the end of round 6, and only ‘slow’ elutions were recovered and carried forward from round 7 onward. The recovered RNA at the end of each round was pooled and passed through a YM-30 spin column to remove excess FAD before RT–PCR. PCR amplification was carried out using 15 cycles of 30 seconds at 94 °C, 60 seconds at 37 °C and 45 seconds at 72 °C with a final 5 minutes at 72 °C after the 15th cycle. RNA for the next selection round was transcribed in vitro from these DNA templates and gel purified as described above. Cherenkov counting of all fractions, including the selection columns, was measured in a scintillation counter, and the percent of eluted RNA was calculated (Extended Data Fig. 1b). During PCR amplifications after rounds 7 and 8, the RNA pool was mutagenized following the protocol of Bartel and Szostak^50^. Increased stringency was applied by increases in wash volumes to 4 ml at round 9 and 20 ml for rounds 10–12. The percent of RNA eluted each round is shown in Extended Data Fig. 1c. After 12 rounds, the binding RNAs were reverse transcribed, PCR amplified and cloned by ligating the PCR product into the pCR2.1-TA vector and transforming into TOP10F′ cells. Thirty-one plasmids were sequenced, and four candidate aptamers (12.2, 12.29, 12.4 and 12.8; Supplementary Table 1) were used for initial testing.

### In-line probing of aptamers with FAD and FADH_2_

Candidate aptamers were tested for FAD and FADH_2_ binding via in-line probing^51,52^. RNAs were radiolabeled with 1 U µl^−1^ of T4 polynucleotide kinase and 10 µCi µl^−1^ of γ-^32^P ATP at 37 °C. Labeled RNAs were first denatured at 85 °C for 3 minutes, followed by incubation in 1× TKNCM buffer pH 8.0 at room temperature for 5 minutes. FAD was added at different concentrations, and samples were incubated at 37 °C for 12 hours. To generate FADH_2_, FAD was incubated in 300 mM Tris pH 8.0 and 175 mM DTT, and reduction was monitored by measuring the color of the solution. For reducing conditions, a layer of mineral oil was placed over the sample to minimize FADH_2_ oxidation by atmospheric oxygen. RNAs were separated by 10% denaturing PAGE. RNA ladders were generated via alkaline or T1 RNase digestion, and unreacted RNA served as a control (Extended Data Fig. 1d). Aptamer 12.29 was truncated to an active 28-nucleotide RNA, designated B2, which was confirmed to bind FAD via in-line probing (Extended Data Fig. 2a). To determine apparent binding constants for FAD and FADH_2_, in-line cleavage signal at U19 of B2 was normalized to full-length unreacted RNA at different concentrations of either FAD or FADH_2_. Data were fitted to one-site binding with shared B_max_ between FAD and FADH_2_ (Extended Data Fig. 2b). The main stem of the B2 aptamer was extended for stability, and the resulting aptamer, named X2B2, was used for all further studies (Extended Data Fig. 2c).

### RNA pre-folding for UV-Vis binding studies and redox assays

RNA oligonucleotides were heat denatured at 85 °C for 2 minutes in a pre-folding buffer containing either 5 mM Tris or HEPES pH 7.5, 15 mM NaCl and 0.1 mM EDTA. Samples were then allowed to fold by incubating at room temperature (17–22 °C) for 15 minutes. Pre-folded aptamers were then introduced to appropriate binding buffers as described below.

### UV-Vis binding assays

UV-Vis-based assays were carried out in 1× TKNM buffer (the same as TKNCM but without Ca^2+^, which we found to be dispensable at the concentrations used) unless otherwise stated. A typical assay contained 50 µM of pre-folded RNA aptamer and 50 µM of flavin (FAD, FMN or Rb). Absorbance was measured in a 10-mm quartz cuvette between 290 nm and 650 nm in 1-nm increments using a NanoDrop 2000c. For aptamer mutants, 40 µM of RNA aptamer and 40 µM of FAD were used. All measurements were normalized so that the maximum absorbance was equal to 1.

### Binding determination of flavin using fluorescence quenching

Fluorescence quenching of FMN upon RNA binding was used to study the divalent metal ion and cobalt hexammine requirements for binding. Each sample contained 100 nM FMN (15 pmol), 3 µM of pre-folded aptamer (450 pmol), 50 mM Tris pH 7.0, 140 mM KCl, 10 mM NaCl and MgCl_2_, MnCl_2_, ZnCl_2_, CaCl_2_ or [Co(NH_3_)_6_]Cl_3_ at concentrations between 1 µM and 100 mM. Another sample containing FMN but no aptamer was measured in parallel to subtract fluorescence quenching by the metal ion. Fluorescence measurements were conducted on a plate reader in black 96-well plates using an excitation wavelength of 450 nm and an emission wavelength of 530 nm, and samples were tested in triplicate. Data were analyzed by calculating the fraction bound using (F − F_0_)/(F_c_ − F_0_), where F was the measured fluorescence of the aptamer–FMN complex at a particular divalent metal ion concentration; F_0_ was the measured fluorescence of only the FMN at a particular divalent metal ion concentration; and F_c_ was the fluorescence of the fully complexed aptamer with FMN and was set to equal the fluorescence of the highest divalent metal ion concentration^53^. Binding curves were generated using specific binding with a variable Hill slope (Y = (B_max_)(X^h^)/(K_d_^h^ + X^h^)) and constraining B_max_ to be equal to 1 (Extended Data Fig. 3c,d).

### Mutational analysis

Based on the initial predicted secondary structure of X2B2 (Fig. 1b), mutants were made by changing either stem (P1 and P2) or loop (L1 and L2) regions. In P1 and P2, mutants were either destructive, where two consecutive nucleotides were changed to disrupt predicted base pairing, or constructive, which mutated opposing nucleotides to restore base pairing with a different identity than the parent aptamer. These mutants tested whether it was the sequence identity or the presence of the base pair that was important for binding. For L1 and L2, individual nucleotides were changed to uracil (or adenosine if the parent nucleotide was already uracil). Mutants were analyzed by UV-Vis as described above, and the absorbance shift relative to free FAD was calculated to yield Δλ_max_ (Supplementary Table 2).

### Native PAGE analysis of mutant aptamers

RNA aptamers (300 pmol) were pre-folded as described above. Then, 300 pmol of FMN, buffer (final concentration of 90 mM Tris pH 8.0, 70 mM KCl, 5 mM NaCl and 15 mM MgCl_2_) and glycerol (final concentration of 10%) were then added to each sample. The gel was prepared with 12% acrylamide, 90 mM Tris pH 8.0, 70 mM KCl, 5 mM NaCl and 15 mM MgCl_2_ and was pre-run at 100 V for 2 hours at room temperature. Samples were loaded, and the gel was run at 100 V for 4.5 hours at room temperature, with frequent monitoring to maintain a temperature of ~24 °C for the entirety of the run. RNAs were imaged on the gel using UV shadow imaging with a UV lamp at 254 nm and a TLC plate with a fluorescent dye. FMN was imaged using FMN fluorescence with a UV lamp at 365 nm. Images were overlayed for analysis (Extended Data Fig. 4).

### ITC

ITC experiments were carried out by titrating FMN, FAD or Rb into X2B2 or X2B2-C14U in matching buffer at 30 °C in 26 injections with 400-second spacing time on a VP-ITC (MicroCal, GE Healthcare). RNA and flavin samples were each prepared separately in 10 mM Tris-HCl pH 7.5 and 20 mM MgCl_2_. Concentrations of each are given as insets in thermograms in Extended Data Fig. 5. Heat-of-dilution titrations were performed by titrating FMN, FAD or Rb into a matching buffer in the same experimental setup. The baseline was corrected by subtracting the heat of dilution, and the data were fitted using ‘one-site’ non-linear least square regression.

### Reduction potential determination

The method for determining the flavin-binding RNA aptamer reduction potentials was adapted from the xanthine–xanthine oxidase reducing system developed by Massey^31,32^. Assays were conducted in a sealable quartz cuvette with a septum and a low flow rate of argon flowing through the headspace via a needle punctured through the septum. Reactions contained 100 mM HEPES pH 7.5, 200 mM KCl, 15 mM MgCl_2_, 700 µM xanthine, 30 µM methyl viologen, 20 µM reference dye, 20 µM flavin, 20 µM pre-folded RNA aptamer, 5 mM glucose, 75 µg ml^−1^ (1.86 U ml^−1^) of glucose oxidase and 20 µg ml^−1^ (44.4 U ml^−1^) of catalase. The glucose, glucose oxidase and catalase created an anaerobic environment by removing molecular oxygen dissolved in solution^54^. The reaction was initiated by anaerobic addition of xanthine oxidase (10–30 µg ml^−1^, 1.5–4.5 U ml^−1^) using a syringe, and the sample was mixed. The amount of xanthine oxidase was kept low to ensure that the reduction of both the flavin–aptamer complex and the reference dye were at equilibrium throughout the assay. UV-Vis absorbance between 290 nm and 750 nm was collected every 1 minute for 120–180 minutes using an Agilent Cary 60 UV-Vis spectrophotometer.

Reduction potentials for free flavins and for aptamer–flavin complexes were calculated by measuring the oxidized-to-reduced ratios for each assay component and assuming that both the flavin and the reference dye were 100% oxidized for the first measurement and 100% reduced when the spectra no longer changed. The percent oxidized for each species was determined by the change in absorbance for the opposing species’ isosbestic point. For assays with AQS, the AQS was measured at 335 nm (flavin isosbestic point), and the flavins and aptamer–flavin complexes were measured at 355 nm (AQS isobestic point). For assays with phenosafranin, the phenosafranin was measured at 540 nm, and the aptamer–flavin complex was measured at 456 nm. Contributions from phenosafranin were subtracted from this measurement. The Nernst concentration terms were then plotted against each other, and a linear best-fit line was determined based on the plotted data with the *y-*intercept (b) being equal to the difference in reduction potential between the flavin and the reference dye (b = E_m,F_ − E_m,D_) in millivolts^55^. All samples were run in triplicate, and the reported reduction potentials were the averages of the triplicates. All E_m_ values are with respect to the standard hydrogen electrode. The absorbance spectrum did not indicate any formation of the semiquinone flavin (λ_max_ ~600 nm) in these assays, indicating that, in all examples, the measured reduction potential was for the 2-electron reduction of the oxidized flavin to the hydroquinone form.

### NMR spectroscopy

X2B2-C14U–FMN and X2B2–FMN complexes were prepared (200–600 µM) and pre-incubated in 10 mM Tris-d11 pD = 7.5 and 20 mM MgCl_2_ at 37 °C for 30 minutes. 2D ^1^H-^1^H NOESY data for fully protonated and site-specifically deuterated samples were collected in D_2_O (CIL; 99.96%) at 308 K and 293 K. 1D and 2D imino proton spectra were collected for X2B2-C14U–FMN in buffer containing 10% D_2_O + 90% H_2_O. Nucleotide-specific ^2^H-labeled samples, including AC-, AG-, GU- and A^2R^U^R^CG-X2B2-C14U, facilitated peak assignments. HNNCOSY data were collected for ^13^C/^15^N-labeled X2B2-C14U–FMN and ^13^C/^15^N-labeled X2B2-C14U–Rb-(dioxopyrimidine-^13^C_4_, ^15^N_2_). All NMR data were collected on a Bruker Avance III 800-MHz spectrometer equipped with TCI cryoprobe (NMR Core, University of Missouri). The NMR data were processed by NMRPipe^56^ and analyzed by NMRViewJ^57^.

### Structure calculation and MD simulations

The X2B2-C14U structure was initially calculated by CYANA using NMR-derived restraints. Standard torsion angle restraints, hydrogen bonding restraints for Watson–Crick base pairs and phosphate distance restraints in RNA helices were employed to maintain the A-form helical geometry in base pairs 1–8, 10–12, 28–30 and 31–38 (refs. ^58,59^). NOE-derived distance restraints were applied for the remaining nucleotides and FMN. Eight structures with the lowest target function were generated and served as initial structures for the MD simulations using Amber 18 (ref. ^60^). Simulated annealing (SA) MD simulations were performed 20 times with the last structure extracted from each trajectory. Then, the total 160 structures were clustered into ten groups using the *k*-means clustering method, and the ten centroid structures were selected as the refined structures sorted in descending order of cluster size.

For SA MD simulations, the RNA molecule was simulated using the RNA.OL3 force field, and the ligand FMN was treated using the GAFF force field generated by the Antechamber package built in Amber. RNA and FMN molecules were solvated in a truncated octahedron periodic box of TIP3P water, and the distance between the edge of the water box and the solute was no less than 12 Å. Moreover, the RNA and FMN system was simulated in roughly 1 M NaCl. After energy minimization, the system was heated from 0 K to 300 K in 10 ps with constant volume and then equilibrated for 10 ps under 300 K and 1 atm pressure. Finally, the SA MD simulation was performed by heating the system to 400 K in 10 ps and then cooling gradually from 400 K to 0 K in about 2.5 ns with constant volume, and the last structure was collected. NOE-derived distance restraints and standard hydrogen bond distance restraints were used during energy minimization, heating, equilibration and SA MD simulation. Eight torsional restraints were employed to keep the nucleotides G15, U18 and A26 coplanar, and positional restraints were used to fix the RNA helical part including the nucleotides 1–12 and 28–38. Statistics of NMR restraints and the calculated structures are summarized in Supplementary Table 5.

### Reporting summary

Further information on research design is available in the Nature Research Reporting Summary linked to this article.

## Online content

Any methods, additional references, Nature Research reporting summaries, source data, extended data, supplementary information, acknowledgements, peer review information; details of author contributions and competing interests; and statements of data and code availability are available at 10.1038/s41589-022-01121-4.

## Supplementary information

Supplementary Information Supplementary Tables 1–5, Supplementary Figs. 1 and 2 and Supplementary Information References.

Reporting Summary

## Data Availability

Depositions for the X2B2-C14U–FMN structure including atomic coordinates (Protein Data Bank: 7RWR) and NMR chemical shifts and restraints for structure calculations (Biological Magnetic Resonance Data Bank: 30942). Data associated with these deposits are shown in Figs. 3–5, Supplementary Table 5 and Supplementary Figs. 1 and 2. Source data are provided with this paper.

## Source data

Source Data Extended Data Fig. 1 Unprocessed gel
Source Data Extended Data Fig. 2 Unprocessed gel
Source Data Extended Data Fig. 4 Unprocessed gels
