# Supplementary material for: ﻿An RNA aptamer that shifts the reduction potential of metabolic cofactors
Source: Nat Chem Biol. 2022 Sep 12;18(11):1263–9. doi: 10.1038/s41589-022-01121-4 (PMC9596375; doi:10.1038/s41589-022-01121-4)
Supplement: Supplementary file 1 — Supplementary Tables 1–5, Supplementary Figs. 1 and 2 and Supplementary Information References. [file 41589_2022_1121_MOESM1_ESM.pdf]

nature portfolio

https://doi.org/10.1038/s41589-022-01121-4

Supplementary information

An RNA aptamer that shifts the reduction potential of metabolic cofactors

In the format provided by the authors and unedited

Supplementary Information

An RNA aptamer that shifts the reduction potential of metabolic cofactors

John S. Samuelian[1], Thomas J. Gremminger[2]†, Zhenwei Song[2], Raghav R. Poudyal[2,3]‡, Jun Li[4], Yuanzhe Zhou[4], Seth A. Staller[2]§, Johan A. Carballo[1], Manami Roychowdhury-Saha[5]¶, Shi-Jie Chen[2,4,6], Donald H. Burke[2,3,7,8,*], Xiao Heng[2,*], & Dana A. Baum[1,*]

[1]Department of Chemistry, Saint Louis University; St. Louis, MO, 63103.

[2]Department of Biochemistry, University of Missouri; Columbia, MO, 65211.

[3]Bond Life Sciences Center, University of Missouri; Columbia, MO, 65211.

[4]Department of Physics, University of Missouri; Columbia, MO, 65211.

[5]Department of Chemistry, Indiana University; Bloomington, IN, 47405.

[6]Institute for Data Science and Informatics, University of Missouri; Columbia, MO, 65211.

[7]Department of Biological Engineering, University of Missouri; Columbia, MO, 65211

[8]Department of Molecular Microbiology and Immunology, University of Missouri; Columbia, MO, 65211.

†Current affiliation = KCAS LLC, 12400 Shawnee Mission Parkway, Shawnee, KS, 66216

‡Current affiliation = Pfizer, Biomedicine Design, 610 N Main Street, Cambridge, MA, 02139.

§Current affiliation = Laronde Inc., 325 Vassar Street, Cambridge, MA, 02139

¶Current affiliation = GRAIL, 1525 O'Brien Drive, Menlo Park, CA, 94025

*Co-corresponding authors: dana.baum@slu.edu; hengx@missouri.edu; burkedh@missouri.edu

Table of Contents

Supplementary Table 1 3

Supplementary Table 2 4

Supplementary Table 3 5

Supplementary Table 4 5

Supplementary Table 5 6

Supplementary Fig. 1 7

Supplementary Fig. 2 9

Supplementary Information References........ 10

Supplementary Table 1: Sequences of primers, selection pool, and RNA aptamers

	Sequence[a]
**Primers**	
Transcription Primer[b]	ACGCACGCTG**TAATACGACTCACTATA**
5' Primer [b]	GC**TAATACGACTCACTATA**GGGAAAAGCGAATCATACACAAGA
3' Primer	TATGGAATTAAATACCTTATGCCC
**Selection Pool** [b]	GC**TAATACGACTCACTATA**GGGAAAAGCGAATCATACACAAGA-(N_{42})-GGGCATAAGGTATTTAATTCCATA
**Aptamers**	
12.2	GGGAAAAGCGAAUCAUACACAAGAUGUGACGCACUAUCUGAUCGGAAAACGUCAUUGUAUGGCGUGGGCAUAAGGUAUUUAAUUCCAUA
12.4	GGGAAAAGCGAAUCAUACACAAGACGAAUGCGACACACUCCGUCCGACUCCCAUCGUGCUCUGGCGUGGGCAUAAGGUAUUUAAUUCCAUA
12.8	GGGAAAAGCGAAUCAUACACAAGAGUGUGACGCACUUUAUGAUCGGAGGCCGUCAUUGUAUGGCGUGGGCAUAAGGUAUUUAAUUCCAUA
12.29	GGGAAAAGCGAAUCAUACACAAGAGCGAUUGAAACUAAGUCUGUGCUAUGUUCGUUAUCGCCGGUGGGGCAUAAGGUAUUUAAUUCCAUA
B2	GGAAAGAGCGAUUGAAACUAAGUUCUUCC
X2B2	GGAUACACAAGAGCGAUUGAAACUAAGUCUGUGUAUCC
Ftest1[c]	GGGCAUAAGGUAUUUAAUUCCAUACAAGUUUACAAGAAAGAUGCA
Neg[d]	GGAACUGCGAUCUAGUGAGACUGACUCGUGAUCGGA

[a]Aptamers B2 and X2B2 are both aligned with their parent aptamer, 12.29. [b]Bold region is promoter for T7 RNA polymerase. [c]ref 28. [d]RNA Ligation product from ref [61]. All sequences are shown 5' – 3'.

Supplementary Table 2: Mutant sequences of X2B2 and UV-Vis spectral data.

Name*	λ_{max}	$\Delta\lambda_{max}$	Sequence (**Red** indicates changes from parental aptamer)
X2B2	456	8	GGAUACACAAGAGCGAUUGAAACUAAGUCUGUGUAUCC
**P2 Mutations**[a]			
X2B2-D1	449	1	GGAUACACAAGAGCGA**AC**GAAACUAAGUCUGUGUAUCC
X2B2-C1	448	0	GGAUACACAAGAGCGA**AC**GAAACUA**GU**UCUGUGUAUCC
X2B2-D2	452	4	GGAUACACAAGAGC**AC**UUGAAACUAAGUCUGUGUAUCC
X2B2-C2	451	3	GGAUACACAAGAGC**AC**UUGAAACUAAG**GU**UGUGUAUCC
**P1 Mutations**			
X2B2-D3	453	5	GGAUACA**GU**AGAGCGAUUGAAACUAAGUCUGUGUAUCC
X2B2-C3	453	5	GGAUACA**GU**AGAGCGAUUGAAACUAAGUC**AC**UGUAUCC
X2B2-D4	454	6	GGAUA**GC**CAAGAGCGAUUGAAACUAAGUCUGUGUAUCC
X2B2-C4	455	7	GGAUA**GC**CAAGAGCGAUUGAAACUAAGUCUG**GC**UAUCC
**L1 Mutations**			
X2B2-A10U	453	5	GGAUACACA**U**GAGCGAUUGAAACUAAGUCUGUGUAUCC
X2B2-G11U	448	0	GGAUACACAA**U**AGCGAUUGAAACUAAGUCUGUGUAUCC
X2B2-A12U	448	0	GGAUACACAAG**U**GCGAUUGAAACUAAGUCUGUGUAUCC
X2B2-G13U	449	1	GGAUACACAAGA**U**CGAUUGAAACUAAGUCUGUGUAUCC
X2B2-C14U	458	10	GGAUACACAAGAG**U**GAUUGAAACUAAGUCUGUGUAUCC
**L2 Mutations**			
X2B2-G19U	450	2	GGAUACACAAGAGCGAUU**U**AAACUAAGUCUGUGUAUCC
X2B2-A20U	448	0	GGAUACACAAGAGCGAUUG**U**AACUAAGUCUGUGUAUCC
X2B2-A21U	449	1	GGAUACACAAGAGCGAUUGA**U**ACUAAGUCUGUGUAUCC
X2B2-A22U	450	2	GGAUACACAAGAGCGAUUGAA**U**CUAAGUCUGUGUAUCC
X2B2-C23U	450	2	GGAUACACAAGAGCGAUUGAAA**U**UAAGUCUGUGUAUCC
X2B2-U24A	454	6	GGAUACACAAGAGCGAUUGAAAC**A**AAGUCUGUGUAUCC
X2B2-A25U	450	2	GGAUACACAAGAGCGAUUGAAACU**U**AGUCUGUGUAUCC
**Mutations based on NMR structure**			
X2B2-C14U-U24A	449	1	GGAUACACAAGAG**U**GAUUGAAAC**A**AAGUCUGUGUAUCC
X2B2-ΔA9	450	2	GGAUACAC**-**AGAGCGAUUGAAACUAAGUCUGUGUAUCC
X2B2-C14U-ΔA9	456	8	GGAUACAC**-**AGAG**U**GAUUGAAACUAAGUCUGUGUAUCC
X2B2-ΔC14	449	1	GGAUACACAAGAG**-**GAUUGAAACUAAGUCUGUGUAUCC
X2B2-C14A	453	5	GGAUACACAAGAG**A**GAUUGAAACUAAGUCUGUGUAUCC
X2B2-C14G	450	2	GGAUACACAAGAG**G**GAUUGAAACUAAGUCUGUGUAUCC
X2B2-BT1	453	5	GGAUACA**AAC**GAGCGAUUGAAACUAAGUC**GU**UGUAUCC
X2B2-C14U-BT1	457	9	GGAUACA**AAC**GAG**U**GAUUGAAACUAAGUC**GU**UGUAUCC
X2B2-C14U-dG13†	458	10	GGAUACACAAGAdG**U**GAUUGAAACUAAGUCUGUGUAUCC
X2B2-C14U-dU18†	455	7	GGAUACACAAGAG**U**GAUdUGAAACUAAGUCUGUGUAUCC
X2B2-C14U-dG13-dU18†	455	7	GGAUACACAAGAdG**U**GAUdUGAAACUAAGUCUGUGUAUCC

$\Delta\lambda_{max}$ is with respect to free FAD λ_{max} = 448 nm

*Aptamer names that are italicized and in red had $\Delta\lambda_{max}$ > 6 nm and are considered to have full FAD/FMN binding compared to the parent aptamer.

[a]Mutations in P1, P2, L1, and L2 based on predicted secondary structure shown in Fig. 1b and Extended Data Fig. 2c.

† Only tested with FMN.

Supplementary Table 3. Thermodynamic parameters from ITC experiments

Aptamer	Flavin	N* (Stoichiometry)	K_d (nM)	ΔH (kcal/mol)	$-T\Delta S$ (kcal/mol)
X2B2	FAD	1.3 ± 0.1	7244 ± 398	-16.8 ± 0.2	9.7 ± 0.2
	FMN	1.1 ± 0.1	1414 ± 97	-19.2 ± 0.8	11.1 ± 0.7
	Rb	1.2 ± 0.1	5333 ± 722	-18.6 ± 1.3	11.3 ± 1.3
X2B2-C14U	FAD	1.0 ± 0.1	818 ± 32	-16.9 ± 0.6	8.5 ± 0.6
	FMN	1.0 ± 0.1	243 ± 28	-16.7 ± 0.9	7.5 ± 0.8
	Rb	1.0 ± 0.1	885 ± 85	-16.4 ± 1.2	8.0 ± 1.2

Data shown is mean ± SD of n=3 independent experiments

*Stoichiometry ratio is $(\text{Flavin})^N : (\text{RNA})^1$

Supplementary Table 4. Thermodynamic calculations from redox assays

Flavin	Aptamer	ΔE_m (mV)	$\Delta\Delta G$ (J/mol)	$K_{d,R} / K_{d,O}$
FAD	X2B2	-11	2122	2.4
	X2B2-C14U	-24	4631	6.6
FMN	X2B2	-22	4245	5.6
	X2B2-C14U	-40	7719	23.3
Rb	X2B2	-14	2701	3.0
	X2B2-C14U	-25	4824	7.1

$\Delta\Delta G = -zF\Delta E_m$

$K_{d,R} / K_{d,O} = 1/\exp(-\Delta\Delta G/RT)$

Supplementary Table 5. Statistics of NMR restraints and the calculated structures

Cyana*	
NOE-derived restraints	238
Intra-residue	42
Sequential	99
Long range |i-j|>1	25
H-bond restraints	72
NOE restraints/residue	6.1
Torsion angles	120
Target function ($Å^2$)	1.3 ± 0.2
Upper distance viol. ($Å^2$)	0.04 ± 0.003
Lower distance viol. ($Å^2$)	0.002 ± 0.0005
Sum VDW viol. ($Å^2$)	3.1 ± 0.3
RMSD (Å)	9.2 ±1.0
Amber	
RMSD (Å)	2.4 ± 0.6
MolProbity analysis†	
Clashscore	1.57, 99th percentile (2)
Probably wrong sugar pucker (%)	0 (0)
Bad backbone conformation	6 (15.4)
Bad bonds (%)	0 (0)
Bad angles (%)	8 (0.57)

*Statistics of 10 lowest energy models.
†The structures refined by MD simulations were evaluated using the MolProbity Server (version 4.2)[62,63].

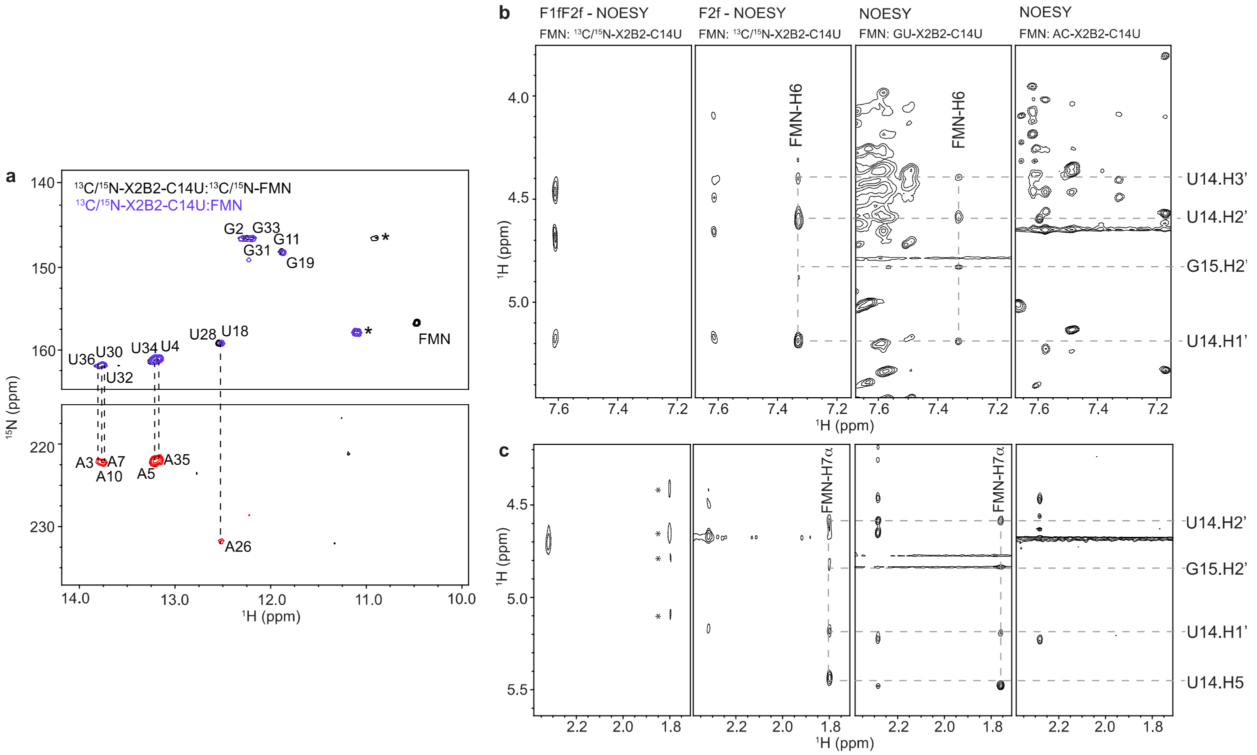

Supplementary Fig. 1: Identification of U18•A26 Hoögsteen pair and NOEs between FMN and X2B2-C14U. a, Portions of the HNN-TOCSY spectra collected for ^{13}C/^{15}N-X2B2-C14U mixing with either unlabeled FMN (blue) or ^{13}C/^{15}N- riboflavin-(dioxopyrimidine-^{13}C4, ^{15}N2) (black) are shown. Cross correlations between U18-H3 and A26-N7 demonstrate the U18•A26 form a Hoögsteen pair. Peaks labeled with asterisk (*) are not observed in imino 2D NOESY spectra and may be from minor RNA conformations. **b**, The intermolecular NOEs between FMN and X2B2-C14U were confirmed by isotope-filtered 2D NMR and nucleotide-specific ^{2}H labeling strategy. Left two panels, portions of F1fF2f-NOESY and F2f-NOESY data collected for ^{13}C/^{15}N-labeled riboflavin mixing with unlabeled FMN (^{12}C/^{14}N) are shown. Right two panels, portions of 2D-NOESY spectra collected for GU-X2B2-C14U (^{2}H-A, C) and AC-X2B2-C14U (^{2}H-G, U) mixing with FMN are shown. (The letters “GU” and “AC” in these RNA species denote the nucleosides containing ^{1}H, which is visible by NMR.) The intermolecular

NOEs between FMN-H6 and X2B2-C14U atoms are labeled. **c**, Portions of F1fF2f-NOESY, F2f-NOESY, FMN:GU-X2B2-C14U and FMN:AC-X2B2-C14U spectra. The intermolecular NOEs between FMN-H7α and X2B2-C14U atoms are labeled. Artifact peaks arising from *t1* noise are labeled with asterisk (*).

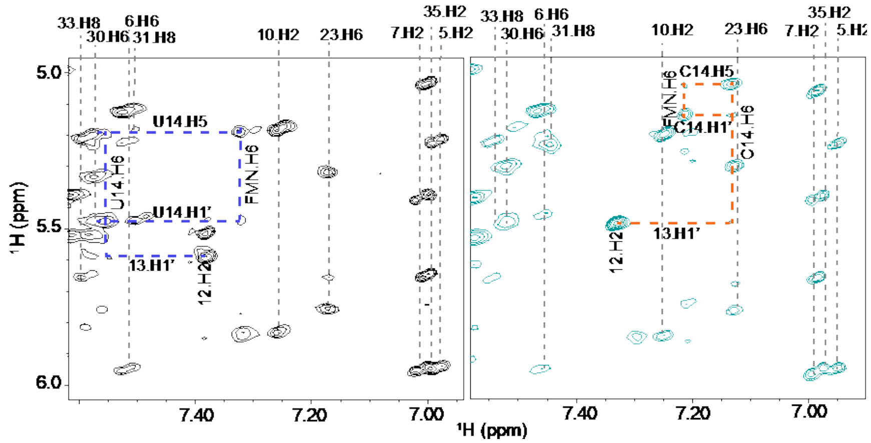

Supplementary Fig. 2. Portion of the 2D NOESY spectra of X2B2-C14U/FMN and X2B2/FMN. The chemical shifts and NOE patterns for most residues remain the same in both spectra (gray dashed lines). The chemical shift of U14 in X2B2-C14U/FMN (left, black) and C14 in X2B2/FMN (right, teal) are different, but both give rise to NOEs to FMN-H6 (thick blue and orange dashed lines, respectively).

Supplementary Information References:

61 Behera, A. K. *et al.* Enhanced deoxyribozyme-catalyzed RNA ligation in the presence of organic cosolvents. *Biopolymers* **99**, 382-391 (2013).

62 Davis, I. W., Murray, L. W., Richardson, J. S. & Richardson, D. C. MOLPROBITY: Structure validation and all-atom contact analysis for nucleic acids and their complexes. *Nucleic Acids Res.* **32**, W615-619 (2004).

63 Davis, I. W. *et al.* MolProbity: All-atom contacts and structure validation for proteins and nucleic acids. *Nucleic Acids Res.* **35**, W375-383 (2007).
