# Supplementary material for: ﻿An RNA aptamer that shifts the reduction potential of metabolic cofactors
Source: Nat Chem Biol. 2022 Sep 12;18(11):1263–9. doi: 10.1038/s41589-022-01121-4 (PMC9596375; doi:10.1038/s41589-022-01121-4)
Supplement: Supplementary file 2 — Reporting Summary [file 41589_2022_1121_MOESM2_ESM.pdf]

nature research

Corresponding author(s): Dana Baum

Last updated by author(s): Jul 12, 2022

Reporting Summary

Nature Research wishes to improve the reproducibility of the work that we publish. This form provides structure for consistency and transparency in reporting. For further information on Nature Research policies, see our Editorial Policies and the Editorial Policy Checklist.

Statistics

For all statistical analyses, confirm that the following items are present in the figure legend, table legend, main text, or Methods section.

n/a	Confirmed	
☐	☒	The exact sample size (*n*) for each experimental group/condition, given as a discrete number and unit of measurement
☐	☒	A statement on whether measurements were taken from distinct samples or whether the same sample was measured repeatedly
☒	☐	The statistical test(s) used AND whether they are one- or two-sided<br>*Only common tests should be described solely by name; describe more complex techniques in the Methods section.*
☒	☐	A description of all covariates tested
☐	☒	A description of any assumptions or corrections, such as tests of normality and adjustment for multiple comparisons
☐	☒	A full description of the statistical parameters including central tendency (e.g. means) or other basic estimates (e.g. regression coefficient) AND variation (e.g. standard deviation) or associated estimates of uncertainty (e.g. confidence intervals)
☒	☐	For null hypothesis testing, the test statistic (e.g. *F*, *t*, *r*) with confidence intervals, effect sizes, degrees of freedom and *P* value noted<br>*Give P values as exact values whenever suitable.*
☒	☐	For Bayesian analysis, information on the choice of priors and Markov chain Monte Carlo settings
☒	☐	For hierarchical and complex designs, identification of the appropriate level for tests and full reporting of outcomes
☒	☐	Estimates of effect sizes (e.g. Cohen's *d*, Pearson's *r*), indicating how they were calculated

Our web collection on statistics for biologists contains articles on many of the points above.

Software and code

Policy information about availability of computer code

Data collection	No computer code was used for data collection.
Data analysis	NMR data was processed by NMRPipe and analyzed by NMRViewJ. Structure calculation was done using CYANA. Molecular dynamics simulations were done using Amber 18 and refined structures were evaluated using MolProbity Server version 4.2. Microsoft Excel 365 and GraphPad Prism 9 were used for spectral data analysis.

For manuscripts utilizing custom algorithms or software that are central to the research but not yet described in published literature, software must be made available to editors and reviewers. We strongly encourage code deposition in a community repository (e.g. GitHub). See the Nature Research guidelines for submitting code & software for further information.

Data

Policy information about availability of data

All manuscripts must include a data availability statement. This statement should provide the following information, where applicable:

- Accession codes, unique identifiers, or web links for publicly available datasets
- A list of figures that have associated raw data
- A description of any restrictions on data availability

Depositions for the X2B2-C14U/FMN structure including atomic coordinates (PDB: 7RWR) and NMR chemical shifts and restraints for structure calculations (BMRB: 30942). Data associated with these deposits are shown in Figure 3, Figure 4, Figure 5, Supplementary Table 5, Supplementary Figure 1, and Supplementary Figure 2.

Field-specific reporting

Please select the one below that is the best fit for your research. If you are not sure, read the appropriate sections before making your selection.

☒ Life sciences ☐ Behavioural & social sciences ☐ Ecological, evolutionary & environmental sciences

For a reference copy of the document with all sections, see nature.com/documents/nr-reporting-summary-flat.pdf

Life sciences study design

All studies must disclose on these points even when the disclosure is negative.

Sample size	No statistical methods were used to predetermine sample size. For UV-vis assays, ITC assays, and the majority of the reduction potential determination assays, n = 3 was selected to provide sufficient statistical power. In a limited number of reduction potential determination assays, n = 2 was used due to limited availability of material, but still provided reproducible results.
Data exclusions	No data were excluded from the analysis.
Replication	For UV-vis, ITC, and reduction potential determination assays, experiments were independently replicated as listed in the corresponding figures. Results were in good agreement and were considered reproducible. In some cases, representative plots are shown.
Randomization	Allocating samples into experimental groups was not done as this is not relevant to the study.
Blinding	Based on the nature of the study, experiments were not blinded. Experiments contained appropriate controls.

Reporting for specific materials, systems and methods

We require information from authors about some types of materials, experimental systems and methods used in many studies. Here, indicate whether each material, system or method listed is relevant to your study. If you are not sure if a list item applies to your research, read the appropriate section before selecting a response.

Materials & experimental systems

n/a	Involved in the study	
☒	☐	Antibodies
☒	☐	Eukaryotic cell lines
☒	☐	Palaeontology and archaeology
☒	☐	Animals and other organisms
☒	☐	Human research participants
☒	☐	Clinical data
☒	☐	Dual use research of concern

Methods

n/a	Involved in the study	
☒	☐	ChIP-seq
☒	☐	Flow cytometry
☒	☐	MRI-based neuroimaging
