# Supplementary figures and images for: ﻿An RNA aptamer that shifts the reduction potential of metabolic cofactors
Source: Nat Chem Biol. 2022 Sep 12;18(11):1263–9. doi: 10.1038/s41589-022-01121-4 (PMC9596375; doi:10.1038/s41589-022-01121-4)

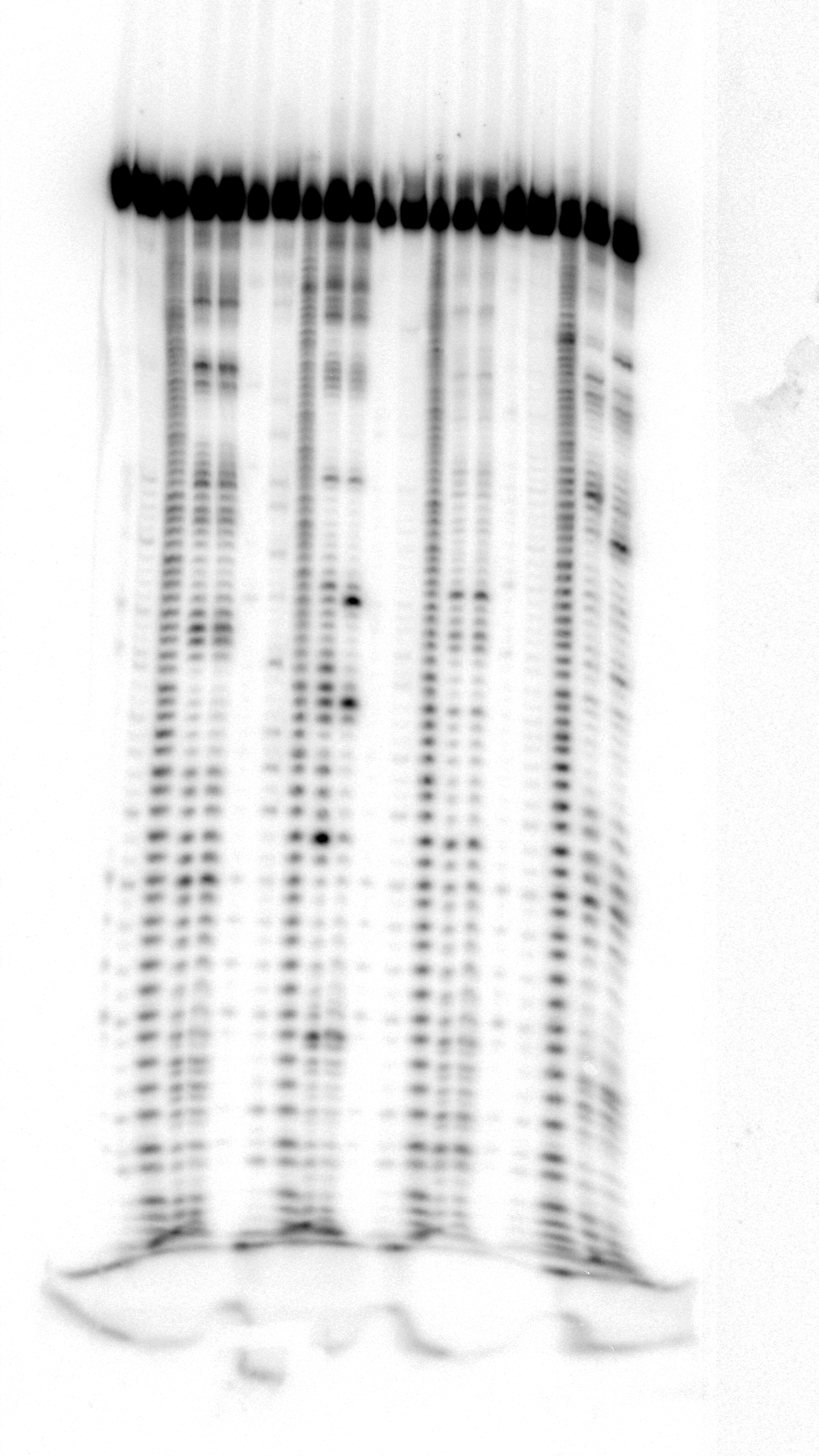

Supplement: Source Data Extended Data Fig. 1 — Unprocessed gel [file 41589_2022_1121_MOESM3_ESM.tif]

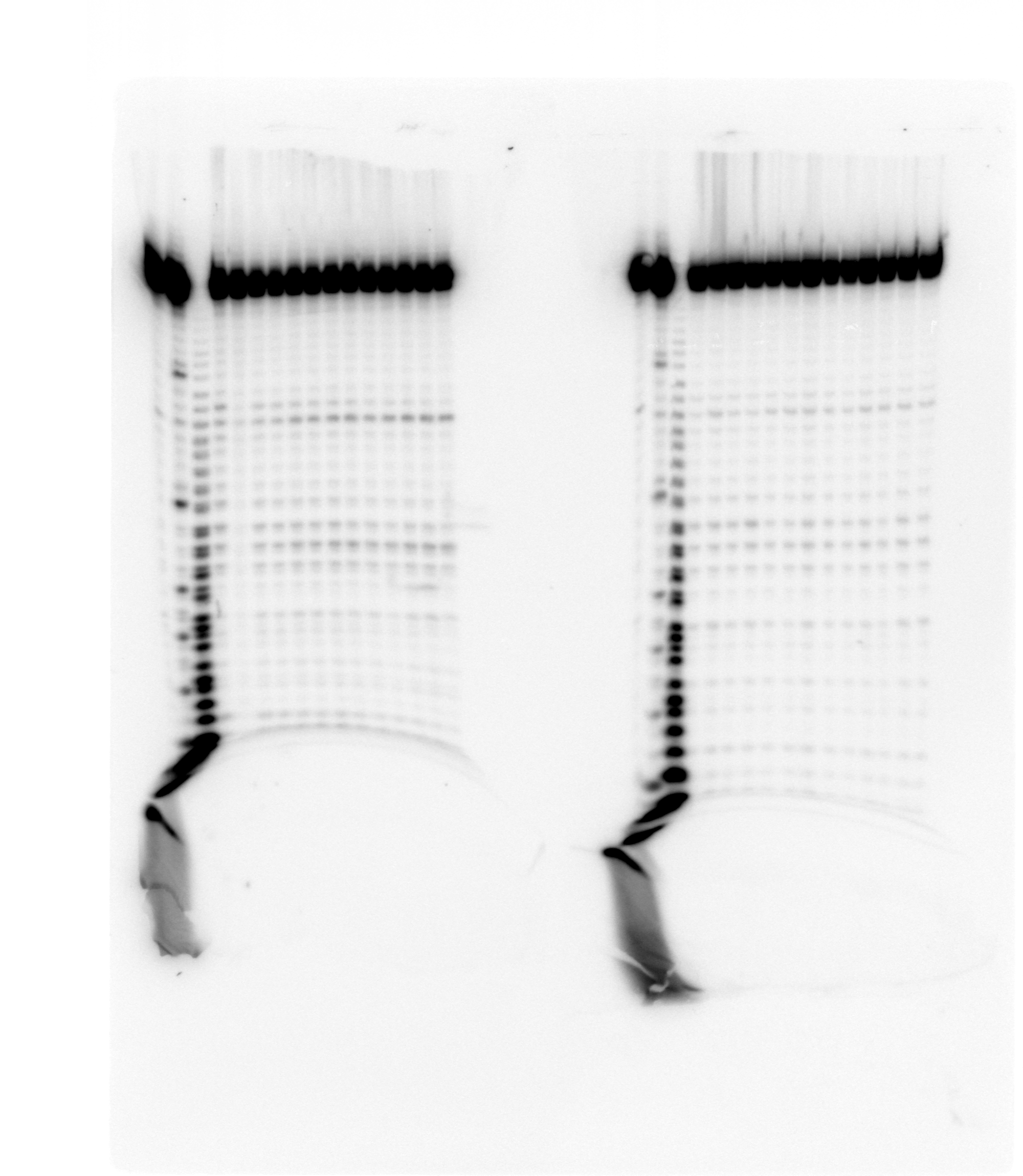

Supplement: Source Data Extended Data Fig. 2 — Unprocessed gel [file 41589_2022_1121_MOESM4_ESM.tif]

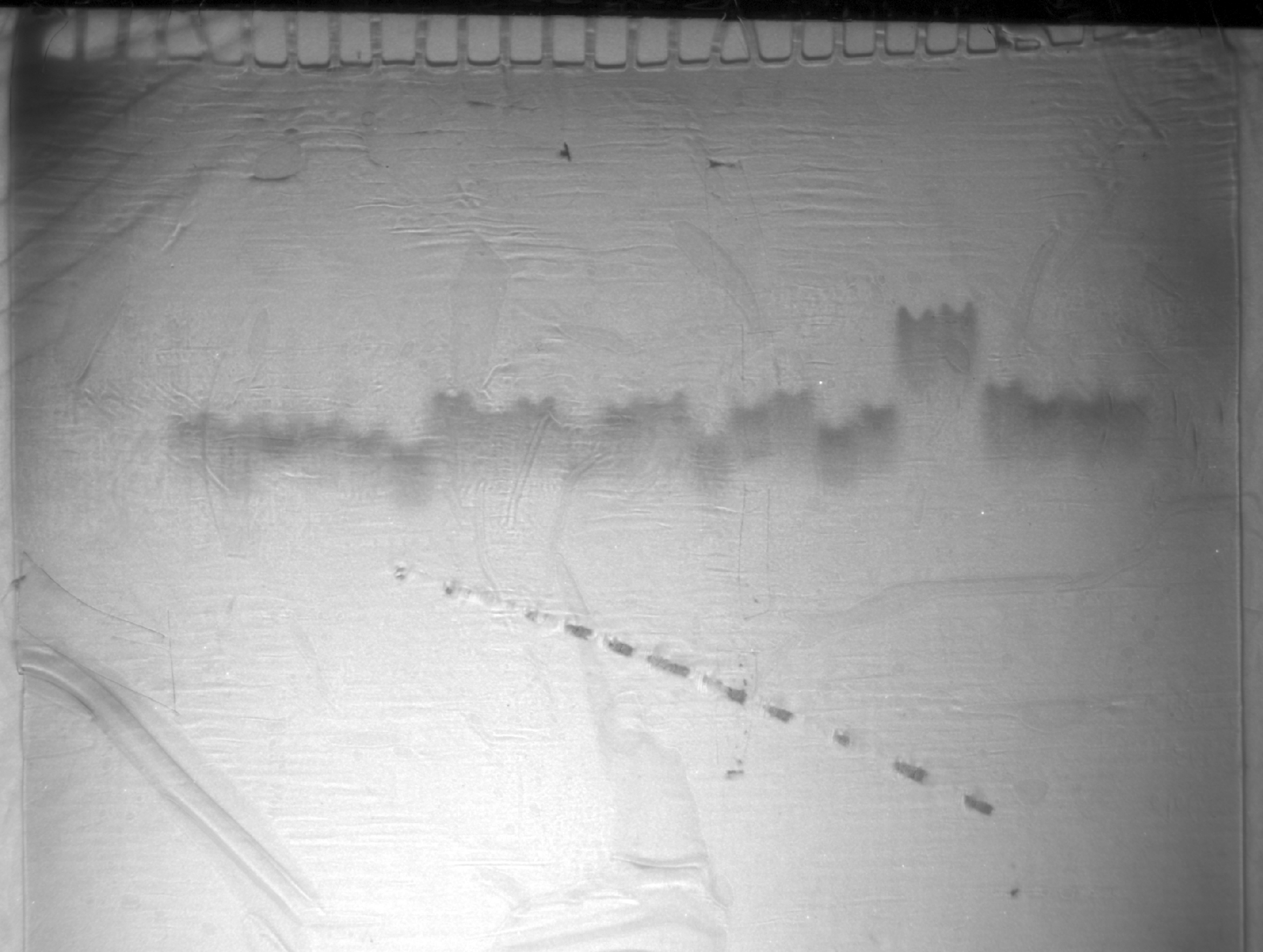

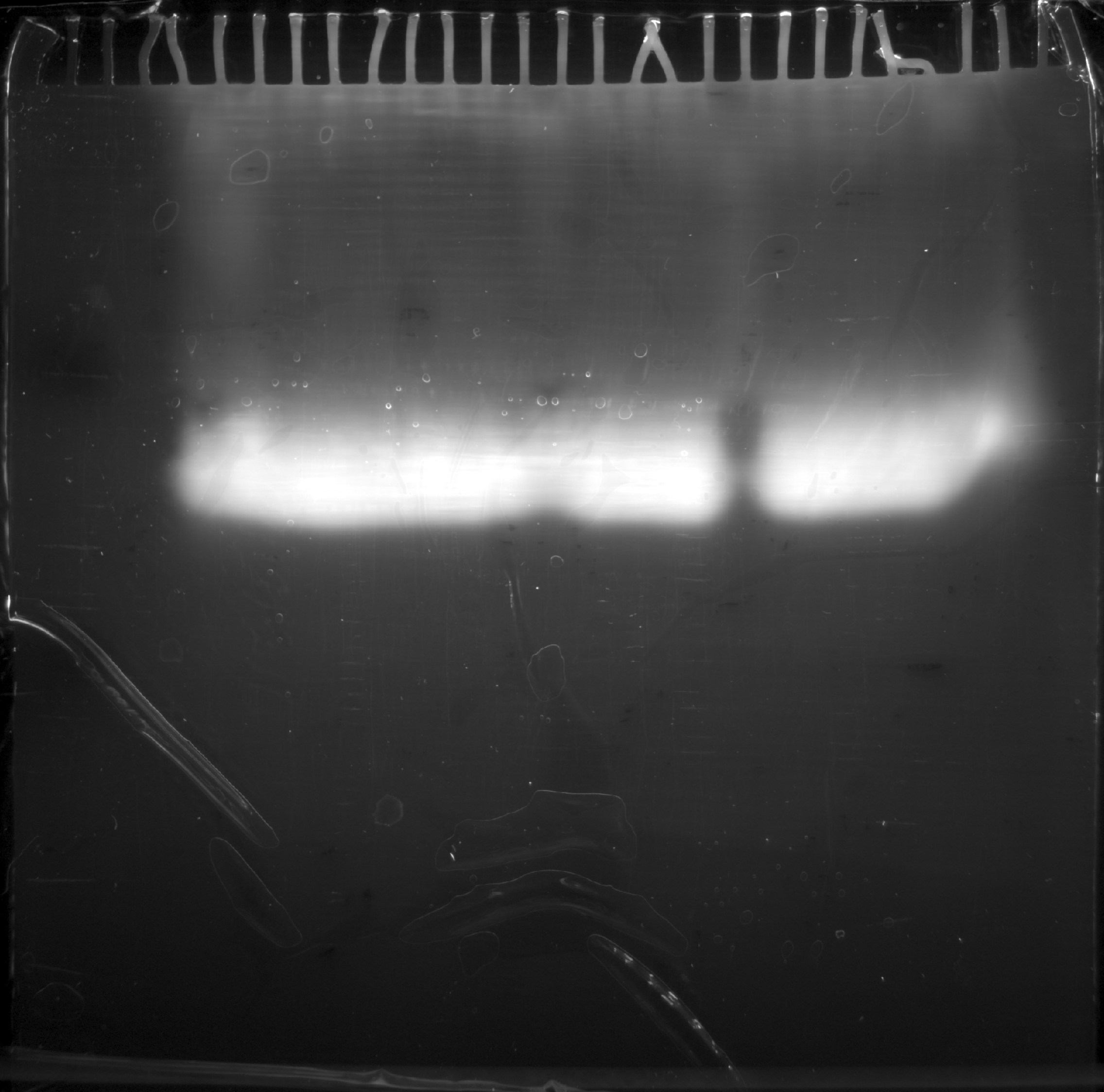

Supplement: Source Data Extended Data Fig. 4 — Unprocessed gels [file 41589_2022_1121_MOESM5_ESM.pdf]
